# Dynamics of microbial communities in Western Antarctic Peninsula waters shaped primarily by the biological interactions

**DOI:** 10.3389/fmicb.2025.1591986

**Published:** 2025-06-25

**Authors:** Mariia Pavlovska, Andrii Zotov, Yevheniia Prekrasna-Kviatkovska, Chandni Sidhu, Artem Dzhulai, Marta Dzyndra, Evgen Dykyi

**Affiliations:** ^1^State Institution National Antarctic Scientific Center, Kyiv, Ukraine; ^2^State Institution Institute of Marine Biology, The NAS of Ukraine, Odesa, Ukraine; ^3^Max Planck Institute for Marine Microbiology, Bremen, Germany; ^4^Graduate School of Oceanography, University of Rhode Island, Narragansett, RI, United States

**Keywords:** bacterioplankton, phytoplankton, Western Antarctic Peninsula, seasonal dynamics, Southern Ocean, Phaeocystis antarctica

## Abstract

Marine Antarctic microbial communities inhabit highly dynamic and extreme environments, characterized by deep vertical mixing, seasonal ice cover, and fluctuating light availability. Understanding the interplay between phytoplankton and bacterioplankton in such systems is critical to elucidate ecosystem function and biogeochemical cycling in the Southern Ocean. The current study presents a comprehensive three-year high-throughput analysis of phytoplankton-bacterioplankton interactions in the waters of Wilhelm Archipelago, elucidating interseasonal and interannual microbial dynamics. The results showed distinct dynamic patterns of microbial taxonomic structure and functional repertoire with heterotrophic phytoplankton-associated bacteria (e.g., *Polaribacter, Yoonia, Sulfitobacter, Amylibacter*, and gammaproteobacterial clade SAR92) dominating in spring and summer, and oligotrophic and chemolithoautotrophic taxa (*Polaromonas* and *Paraglaciecola*) prevailing in autumn. Positive correlations were detected between *Bacillariophyceae, Coccolithophyceae*, and *Dinophyceaea* with *Sulfitobacter* and *Yoonia*, emphasizing their association with phytoplankton abundance. Indirect functional predictions using the PICRUSt2 pipeline demonstrated seasonal shifts in bacterioplankton metabolic potential. Bacterial genes encoding carbohydrate degradation and sulfatases, crucial for algal sulfated polysaccharide breakdown, were most abundant during phytoplankton development, while DMSP demethylation genes peaked in summers of 2019 and 2020, following ice retreat and mass-development of *Phaeocystis antarctica* (*Coccolithophyceae*). Additionally, elevated uric acid degradation genes suggest an ornithogenic influence from the expanding penguin colony on nitrogen cycling within the marine ecosystem. These findings highlight the pivotal role of seasonal phytoplankton dynamics in structuring bacterioplankton communities and provide novel insights into microbial-mediated biogeochemical processes in the Southern Ocean.

## 1 Introduction

The Southern Ocean is a large and heterogeneous system with distinct seasonality, encompassing highly productive coastal waters and offshore regions, where primary productivity is primarily limited by the availability of nutrients and microelements (Signori et al., 2014). Additionally, the marine Antarctic region is characterized by the most pronounced warming and ice cover reduction on the planet, making it an ideal location for monitoring the impact of climate change on marine ecosystems (Ducklow et al., 2013; Saba et al., 2014).

Marine Antarctic microbes thrive in unique and extreme environmental conditions, ranging from deep vertical mixing of water masses, the absence of light, dense ice cover, and minimal photosynthetic activity during winter, to the contrasting conditions in summer (Luria et al., 2017). Strong seasonal variation drives the succession of microbial communities, leading to dynamic changes in both their taxonomic and functional structure (Murray et al., 1998; Grzymski et al., 2012; Wilkins et al., 2013). Sea surface temperature and the seasonality of ice cover are considered to be the major environmental factors driving the dynamics of bacterial production and community composition (Ducklow et al., 2013), while nutrients availability and salinity influence microbial communities at small scales (Singh et al., 2015; Cordone et al., 2022) Heterotrophic and photoheterotrophic microorganisms are predominant in summer bacterioplankton communities (Wilkins et al., 2013; Fuentes et al., 2019; Cordone et al., 2022), while chemolithoautotrophic bacteria dominate during winter (Wilkins et al., 2013; Alonso-Sáez et al., 2011). Shoreline proximity has also been identified as a factor shaping bacterioplankton community composition, with *Roseobacter* (class *Alphaproteobacteria*) and *Zavarzinella* (phylum *Planctomycetes*) being more abundant near the coast (Luria et al., 2014).

Bacterial heterotrophic primary production in coastal waters is regulated by both bottom-up and top-down controls, including the availability of organic carbon supplied by phytoplankton (Seymour et al., 2017; Ferrer-González et al., 2021; Sidhu et al., 2023) and grazing pressure by mixotrophic cryptophytes (Bowman et al., 2017). The interaction between bacterioplankton and phytoplankton intensifies during phytoplankton bloom, fueling the microbial loop and playing a pivotal role in the flux of energy and nutrients (Deppeler and Davidson, 2017). Recent warming trends, leading to a shorter winter ice season in the duration in Western Antarctic Peninsula (WAP) ecosystem and an increased influx of freshwater, have the potential to alter phytoplankton community structure and dissolved organic carbon availability. These changes, in turn, could impact bacterioplankton diversity and functional dynamics (Kim and Ducklow, 2016).

Antarctic marine microbial communities have been studied across various regions of the Southern Ocean. In coastal waters near British Rothera Station, a shift from *Alpha-* and *Gammaproteobacteria* to *Cytophaga–Flavobacterium–Bacteroides* (CFB) (*Bacteroidetes*) was observed during the summer transition from stratified to mixed waters (Piquet et al., 2011). Seasonal, vertical and geographic variations were shown for eukaryotic, bacterial and archaeal communities along the WAP. Eukaryotic communities displayed the most pronounced differences between the southern and the northern sites, while bacterial communities showed greater variation with depth, and archaeal communities exhibited more seasonal fluctuations (Luria et al., 2014).

The coupling of bacterial production, richness and community composition with phytoplankton dynamics was observed near Palmer station. Specifically, *Flavobacteria* and *Rhodobacteraceae* were found to increase in abundance in response to phytoplankton blooms (Luria et al., 2016). This bacterioplankton-phytoplankton interplay was further explored by Kim et al. (2019) and the link between bacterial taxonomic and functional profiles and different algal bloom phases was established. It was found that *Polaribacter* was dominating at the bloom peak and its potential to degrade chrysolaminarin and xylan from haptophyte *Phaeocystis antarctica* was discovered based on metatranscriptome data (Kim et al., 2019). An increase in bacterioplankton abundance was detected in the Weddell Sea during the austral summer phytoplankton bloom (Piontek et al., 2022). In contrast, low-chlorophyll stations were characterized by highly-interconnected bacterial communities, indicating mutually beneficial interactions during periods of low carbon availability (Piontek et al., 2022). Diatom species were found to correlate with the prevalence of heterotrophic bacterioplankton, such as *Polaribacter*, in coastal regions, while haptophyte blooms promoted the dominance of oligotrophic and mixotrophic microbes in offshore waters during austral summer (Cordone et al., 2022). The functional strategies of Antarctic heterotrophic bacterioplankton have been further explored through metagenome-assembled genomes (MAGs) of key taxa that respond to the influx of phytoplankton-derived organic matter in the Weddell Sea. Distinct genomic mechanisms directed toward dissolved organic matter (DOM) utilization have been identified in members of *Bacteroidota* and *Gammaproteobacteria*, with high transcription of glycoside hydrolases, aminopeptidases, and transporters (Piontek et al., 2024).

Incubation studies of the Southern Ocean bacterioplankton have revealed niche-partitioning among microbes in terms of their preferences for DOM utilization. The genera such as *Sulfitobacter, Colwellia* and *Methylophaga* responded positively to the addition of diatom-derived DOM, while *Polaribacter, Marinobacter*, NAC11–7 (*Roseobacter*) and SAR11 clades showed preferences toward organic matter with lower biological availability, typical of winter conditions (Landa et al., 2018). Additionally, a growth promoting effect of *Roseobacter* and an inhibitory effect of *Flavobacterium* on *Pseudo-nitzschia* was also observed in a culture-based study (Andrew et al., 2022).

Despite existing research, data on the factors shaping the taxonomic and functional composition of the Southern Ocean microbial communities remains limited, mainly to spring and summer seasons (Ozturk et al., 2022). The significant interseasonal and interannual variability of environmental conditions contributes uncertainty to predictions of microbial community dynamics, which in turn limits our understanding of the processes these communities mediate in the most efficient carbon sink on our planet (Castillo et al., 2022). The current study aims to identify patterns in phytoplankton-bacterioplankton interactions and the environmental drivers behind them, based on the high-throughput three-year study in the waters of Wilhelm Archipelago, that combined 16S rRNA gene amplicon sequencing, metabolic function prediction, and contextual environmental data.

## 2 Methods

### 2.1 Bacterioplankton and phytoplankton sampling

**Figure 1:**
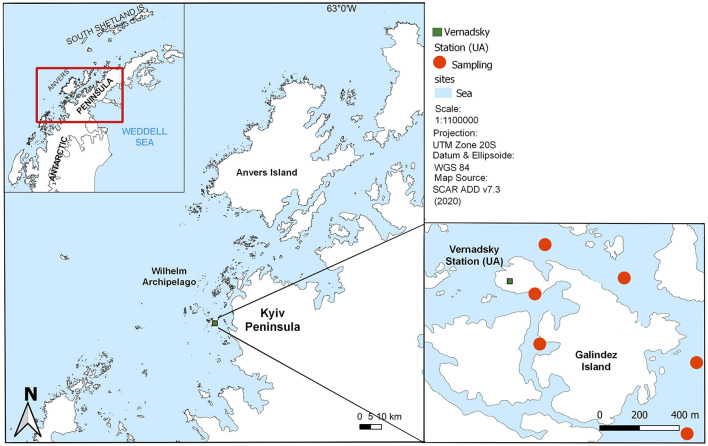
Marine monitoring stations near Galindez island (Wilhelm Archipelago).

Seawater samples were collected near Galindez island (Wilhelm Archipelago, Western Antarctic Peninsula) during three consecutive Ukrainian Antarctic Expeditions from 2019 to 2021, covering the period from February to April (Figure 1, Supplementary Table 1). Additional sampling was conducted between October and January in 2019 and 2020. This ensured that our sampling campaign spanned Antarctic spring, summer and autumn. Samples were collected at varying distances from the coast at Stations 1, 3 and 5 during February to April and at Station 3 between October and January, as most of the water area was inaccessible due to adverse weather conditions and ice cover in spring. In total, 280 surface seawater samples were collected, enabling the analysis of both intra- and interseasonal dynamics of bacterioplankton communities.

Samples of 5 L were collected using the Niskin bottle (Hydro-bios, Altenholz, Germany) and the temperature and salinity profiles at each station were recorded with an RBR Concerto CTD (RBR Ltd., Ottawa, Canada). Seawater was immediately processed for bacterioplankton sampling using vacuum filtration with a Combisart.jet Sartorius pump (Sartorius, Göttingen, Germany) through 0.22 μm PC Millipore filters (Millipore, Biilerica, MA, USA). The filters were preserved in RNAlater (Sigma-Aldrich, St. Louis, MO, USA) solution and stored at −20°C until DNA extraction.

Phytoplankton was concentrated to 30–50 ml with reverse-flow filtration through 1.2 μm filters (Millipore). The samples were fixed with a 50/50 solution of formalin (40%) and glacial acetic acid, at a final formalin concentration of 4%. To further concentrate the samples, slow decantation was applied, reducing the volume to 10–20 ml for visual assessment. Quantitative and taxonomic analysis of phytoplankton was performed using a Zeiss Primo Star microscope at 400–1,000 × magnification (Zeiss, Oberkochen, Germany). Cell counts were calculated in triplicate using a 0.05 ml Nageotte counting chamber. For larger cells, a 1 ml Sedgewick-Rafter counting chamber was used. Wet biomass (mg/m^3^) was calculated using the geometric similarity method as described by (Utermöhl, 1958).

### 2.2 Chlorophyll *a* and hydrochemical parameters estimation

Two-liter (2 L) seawater samples were filtered through 0.45 μm cellulose nitrate filters (Millipore) using a Combisart.jet vacuum pump (Sartorius, Germany). Pigments were extracted from the filters using acidic acetone, and the concentrations of chlorophyll *a* (Chl *a*) and phaeophytin were measured with a Hach Lange DR3900 spectrophotometer (Hach, Loveland, CO, USA).

The concentrations of ammonium (${\text{NH}}_{4}^{+}$), nitrite (${\text{NO}}_{2}^{-}$), nitrate (${\text{NO}}_{3}^{-}$), phosphate (${\text{PO}}_{4}^{3-}$), silicon (Si) and iron (Fe) were determined using the Hach Lange DR3900 spectrophotometer with the following reagent kits, according to the manufacturer's protocol: Nitrogen-Ammonia Reagent Set (Salicylate Method), NitriVer 3, NitraVer 5, PhosVer 3, Silica Reagent Set (High Range) and FerroZine.

### 2.3 DNA extraction, sequencing and data availability

DNA was extracted from PC filters containing bacterioplankton biomass using the Higher Purity Soil DNA Isolation kit (Canvax Biotech, Spain). The concentration and quality of the resulting DNA was assessed with a DeNovix DS-11 FX + spectrophotometer/fluorometer (DeNovix Inc., USA).

The 16S rRNA gene sequencing was performed at Novogen (Novogene Co., Ltd., UK) using the Illumina NovaSeq PE250 platform with 515F-806R barcode universal primers (Caporaso et al., 2011) targeting V4 region. PCR amplification was carried out using Phusion High-Fidelity PCR Mix (New England Biolabs) under the following conditions: an initial denaturation at 98°C for 1 min, followed by 30 cycles of denaturation at 98°C for 10 s, annealing at 50°C for 30 s, and elongation at 72°C for 60 s, with a final elongation step at 72°C for 5 min. PCR products were analyzed on a 2% agarose gel and purified using the Qiagen Gel Extraction Kit (Qiagen, Germany) prior to library preparation with Illumina NEB Next Ultra DNA Library Preparation Kit. The Qubit 2.0 fluorometer (Thermo Scientific, USA) and Agilent Bioanalyzer 2100 (Agilent, USA) system were used to assess the library quality before sequencing. Sequencing data generated within the framework of this study have been deposited in the NCBI database under accession number PRJNA1234644.

### 2.4 Bioinformatic workflow and statistical analysis

The resulting sequences were merged and reoriented in the 5′-3′ direction using FLASH (v1.2.7) (Magoč and Salzberg, 2011). QIIME2 2019.7 (Bolyen et al., 2019) was employed to remove barcodes, homopolymers, chimeric and short (<150 bp) sequences. Operational Taxonomic Units (OTUs) were inferred through *de novo* clustering at 97% similarity using the UPARSE algorithm (Uparse v7.0.1090, Edgar, 2013). The taxonomic assignment of OTUs was performed based on the Silva SSU Ref NR 99 database (v138) (Quast et al., 2013).

The “Diversity core metrics” plugin in QIIME2 2019.7 was used to calculate the Shannon diversity index and phylogenetic distance (PD) after rarefication to 88126 reads. Data normality was assessed using the Shapiro-Wilk test in R (v.2.15.3) (R Core Development team) and interseasonal and interannual differences in bacterioplankton taxonomic structure were tested using the non-parametric ANOSIM test. A non-metric multidimensional scaling (NMDS) plot based on Bray-Curtis dissimilarity was used for visualization, while Bray-Curtis distance matrix was applied in a constrained ordination (CAP) using the *vegan* package in R (Oksanen et al., 2025). Mantel test with Spearman correlation was conducted to determine the influence of environmental factors (salinity, temperature, Chl *a*, hydrochemical parameters, phytoplankton abundance and biomass) on the variance in microbial community taxonomic structure across the sampling period.

The functional potential of bacterioplankton community structure was predicted with PICRUSt2 pipeline (Phylogenetic Investigation of Communities by Reconstruction of Unobserved States) based on 16S rRNA gene sequencing data using the OTUs clustered at 97% (Douglas et al., 2020). Community structure and differentiation, as well as correlation matrices, were visualized using GraphPad Prism (v10, GraphPad Software, Boston, Massachusetts USA), and Python with the networkX package (Hagberg et al., 2008).

## 3 Results

Seasonal dynamics of bacterioplankton taxonomic and functional profile was observed in line with the changes in phytoplankton community, physical and chemical parameters of the environment. It is important to note that the logistical challenges prevented us from measuring physico-chemical parameters in October 2020, December 2020 and January 2021, which set certain limits to the subsequent analysis.

Interannual and interseasonal dynamics of physical parameters, salinity and temperature (Supplementary Figure 2B) and chemical parameters, Chl *a*, dissolved inorganic nitrogen, phosphorus, ammonium, nitrite, iron and silica (Supplementary Figure 2A) were observed during the survey period. In general, the summer period was accompanied by higher temperatures and lower salinity, with values reaching T = 2.3 °C and S ‰ = 30.0 on February 3^rd^ 2020. Conversely, lower temperature and higher salinity periods were recorded in spring (T = −1.4°C and S ‰ = 34.0 on October 31^st^, 2019) before the ice meltdown and in autumn (T = −0.4°C and S ‰ = 32.4 on October 31^st^, 2019). Si concentration fluctuated with the opposite trend to Chl *a*, dropping to 4.0 mg/L during the haptophyte mass-development in summer 2019 and increasing to 11.0 mg/L early in March 2019, after the bloom collapse (Supplementary Figure 2B). The peaks in iron and dissolved inorganic nitrogen concentration corresponded to the low-chlorophyll periods reaching 6.8 mcg/L and 2.17 mg/L during summer-autumn transition of 2021 (Supplementary Figure 2B).

### 3.1 Phytoplankton dynamics and bacterioplankton community structure

**Figure 2:**
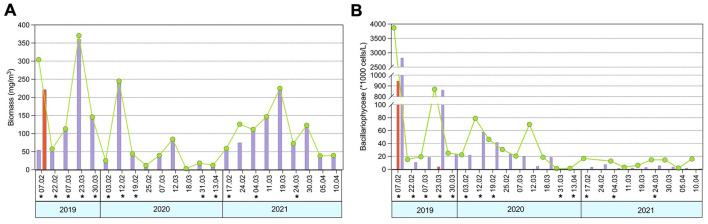
Dynamics of phytoplankton biomass **(A)** and abundance **(B)** during the survey period. Bacterioplankton sampling dates are indicated with asterisk (*).

The three consecutive sampling years represented distinct scenarios in terms of phytoplankton dynamics. The beginning of the sampling period, February 2019, was marked by a more complex phytoplankton community due to the mass development of *Coccolithophyceae* with biomass reaching 221.7 mg/m^3^ on February 7^th^, followed by *Bacillariophyceae*, which peaked at 360.8 mg/m^3^ on March 7^th^ (Figure 2A). In contrast, the following two survey years were dominated exclusively by diatoms, with higher biomass recorded in 2021 (146.2 mg/m^3^ on March 11^th^). Phytoplankton abundance was highest in February–March 2019, with peak values of 2,822.0^*^1,000 cells/L and 946.2^*^1,000 cells/L on February 7^th^ for *Bacillariophyceae* and *Coccolithophyceae*, respectively. A secondary maximum in diatom abundance was observed on March 23^rd^, reaching 218.6^*^1,000 cells/L (Figure 2B). In contrast, the 2020 and 2021 sampling years were characterized by low phytoplankton abundance, which never exceeded 57.92^*^1,000 cells/L, observed on February 12^th^, 2020.

Amplicon sequencing yielded an average of 101,624 non-chimeric sequences per sample, with a minimum of 90,573 in February 2019 and a maximum of 138,517 sequences in March 2019 (Supplementary Table 2). Rarefaction curves indicated that the sequencing depth was sufficient to ensure the reliability of subsequent microbial community structure analysis (Supplementary Figure 1). The Shannon diversity index averaged 4.9, with the highest value of 5.6 observed in October 2020 and the lowest in October 2019 (Supplementary Figure 3A). In contrast, the phylogenetic diversity (PD), which is a measure of diversity based on phylogenetic tree, was highest in February 2019 with an estimate of 140.6, and the lowest in October 2019 with a value of 47.7 (Supplementary Figure 3B).

NMDS analysis revealed interseasonal taxonomic differentiation across all three sampling years. In 2019, two distinct clusters were identified, representing summer and late summer-early autumn seasons (Supplementary Figure 4A). Likewise, in 2020, the bacterioplankton community grouped according to the sampling season, with early summer samples showing taxonomic similarity to spring samples, mid-summer clustering separately and the third cluster formed by late summer-autumn (Supplementary Figure 4B). Similar seasonal differentiation patterns were observed in 2021 (Supplementary Figure 4C). However, the samples from the same seasons in different years clustered separately, suggesting significant inter-annual variation (Supplementary Figure 4D). Statistically significant seasonal differentiation of bacterioplankton communities was supported by the ANOSIM results in 2019 (*R* = 0.5, *p* = 0.002), 2020 (*R* = 0.77, *p* = 0.001), and 2021 (*R* = 0.35, *p* = 0.002).

The bacterioplankton community structure was predominantly represented by classes *Bacteroidia, Alphaproteobacteria, Gammaproteobacteria, Verrucomicrobiae, Actinobacteria* and *Clostridia* (Supplementary Figure 5). *Alphaproteobacteria* were the most abundant in February 2019 and January 2020, accounting for 62% and 57% of the total community, respectively (Supplementary Figure 5). In contrast, *Bacteroidia* constituted a substantial fraction of the spring bacterioplankton community in 2019, accounting 69% in October and 67% in December. In 2021, the majority of the bacterioplankton community was represented by *Gammaproteobacteria*, with their relative abundance fluctuating between 35% and 44% on March 4^th^ and March 11^th^, respectively (Supplementary Figure 5). *Verrucomicrobiae* were notably abundant in autumn 2019, comprising 4.3% of the community, while *Actinobacteria* peaked in late summer 2021, accounting for 8%.

**Figure 3:**
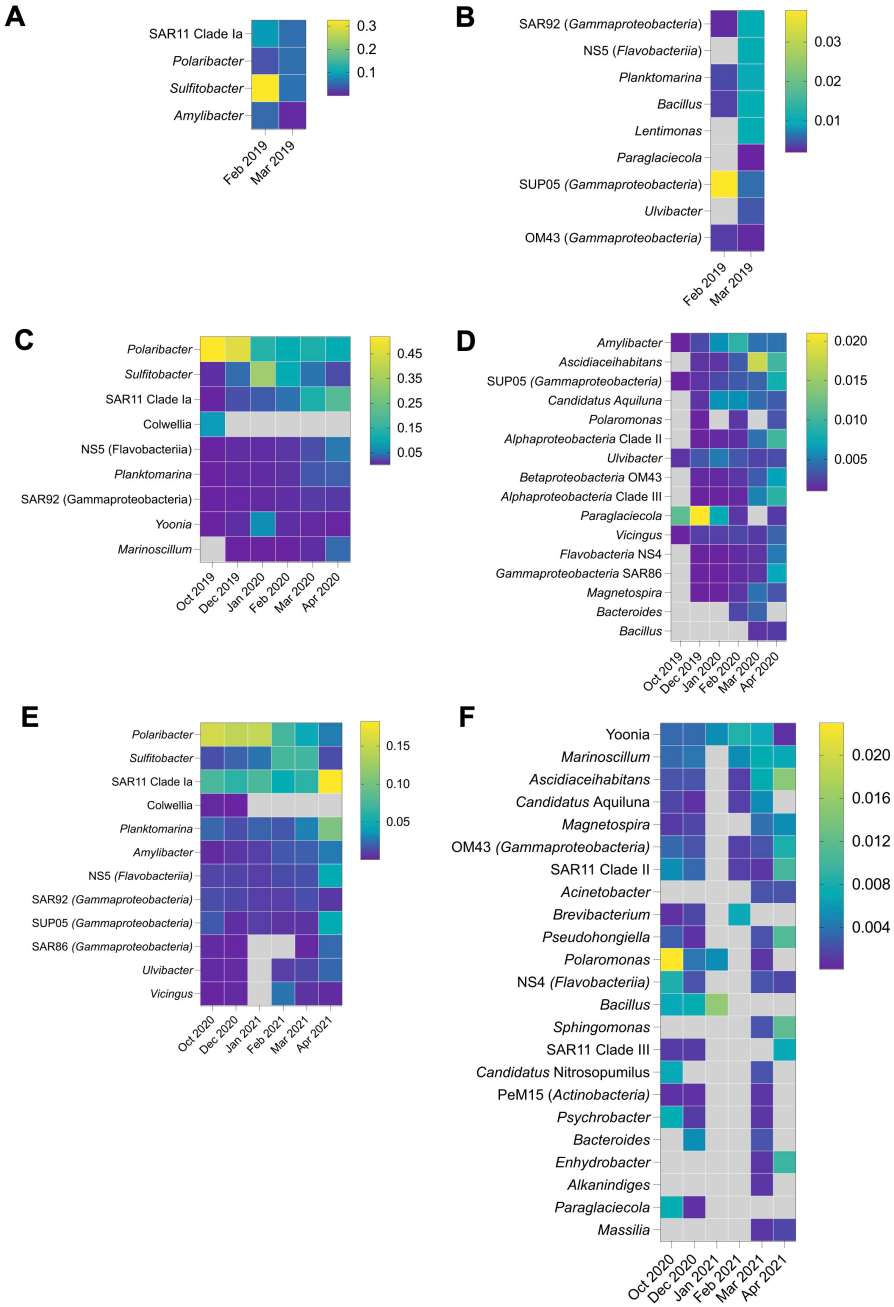
Bacterioplankton taxonomic structure at the genus level in 2019 **(A, B)**, 2020 **(C, D)**, and 2021 **(E, F)**.

The bacterioplankton community in 2019 was characterized by the dominance of fewer genera, whereas a more heterogenous community structure with a greater number of genera in lower relative abundance was observed in 2020 and 2021 (Figures 3A–F). *Sulfitobacter* dominated the late summer community in 2019, with its relative abundance fluctuating from 63% on February 7^th^ to 1.2% on February 23^rd^ (Figure 3A). High taxonomic diversity was observed in autumn 2019, with notable contributions from *Polaribacter* (6% on average), *Sulfitobacter* (5%), SAR11 clade Ia, *Amylibacter*, gammaproteobacterial clade SAR92, flavobacterial clade NS5, *Planktomarina, Bacillus* and *Lentimonas* (each constituting ~1% of the total community) (Figures 3A, B). The spring and early summer bacterioplankton community, preceding the summer of 2020, exhibited strong dominance by *Polaribacter*, which accounted for 52% and 45% of the total community in October 2019 and December 2019, respectively.

Similar to 2019, *Sulfitobacter* dominated the microbial community in summer 2020, averaging 30% relative abundance and decreasing to 2.4% in autumn 2020 (Figures 3C, D). *Polaribacter* remained abundant throughout the sampling period, reaching maximum at 15.4% in October 2020 before the onset of 2020–2021 summer season. *Yoonia* (class *Alphaproteobacteria*) reached a considerable relative abundance of 5.7% exclusively in January. As in 2019, particularly high diversity was observed in autumn 2020, with SAR11 clade Ia dominating at 19.7% in April. Other genera, including *Ascidiaceihabitans*, SAR11 clade II and III (class *Alphaproteobacteria*) and SUP05, OM43, and SAR86 clades (class *Gammaproteobacteria*) each accounting for 1% on average (Figures 3C, D).

Bacterioplankton community in 2021 exhibited a more even taxa distribution. A gradual shift was observed during the summer-autumn period, transitioning from the dominance of heterotrophic taxa associated with phytoplankton exudates to free-living bacteria. Specifically, the relative abundance of *Polaribacter* declined from 14.5% in January to 2.8% in April, while SAR11 clade Ia increased from 2 to 18.3% during the same period (Figure 3E). *Planktomarina*, NS5 and SUP05 clades reached their highest relative abundances of 10.2, 4.5, and 4.5%, respectively, in April 2021.

In contrast to 2019 and 2020, *Sulfitobacter* was more abundant in February and March 2021, reaching 7% (Figure 3E). As in previous years, the autumn bacterioplankton community was characterized by higher diversity compared to summer. Genera such as *Ascidiaceihabitans*, SAR11 clade II, *Pseudohongiella, Sphingomonas* exceeded 1% relative abundance in April 2021 (Figure 3F). Statistically significant differences were observed in both inter- and intraseasonal distribution of certain genera (Supplementary Table 3). The 2019 and 2020 sampling years displayed more taxa with differential seasonal distribution compared to 2021, likely due to the absence of spring 2021 data at the time of analysis. The genera such as *Polaribacter, Bacillus, Amylibacter*, SAR92, *Paraglaciecola* and *Ascidiaceihabitans* exhibited statistically significant variations in relative abundance across spring, summer and autumn in all three sampling years. In contrast, the relative abundances of *Colwellia* and *Pseudoalteromonas* differed only between March and October 2019 (Supplementary Table 3). Additionally, the relative abundance of *Yoonia, Marinoscillum* and SUP05 showed seasonal variations exclusively in 2020, while *Ulvibacter* displayed significant differences between summer and autumn in 2021.

In essence, our analyses suggest that bacterioplankton community dynamics exhibited recurring seasonal patterns, with specific taxa dominating in particular periods, suggesting a degree of predictability in microbial succession. However, notable interannual differences also emerged, reflecting shifts in the timing, magnitude, and composition of phytoplankton development across the three consecutive sampling years.

### 3.2 Bacterioplankton taxonomic structure influenced by varying biotic and abiotic factors across different seasons in the waters of Wilhelm archipelago

The ordination analysis to uncover the biotic and abiotic factors shaping microbial communities dynamics (canonical analysis of principal coordinates (CAP) was performed for samples from February – April of the three consecutive sampling years, as no phytoplankton data was available for spring and early summer seasons.

**Figure 4:**
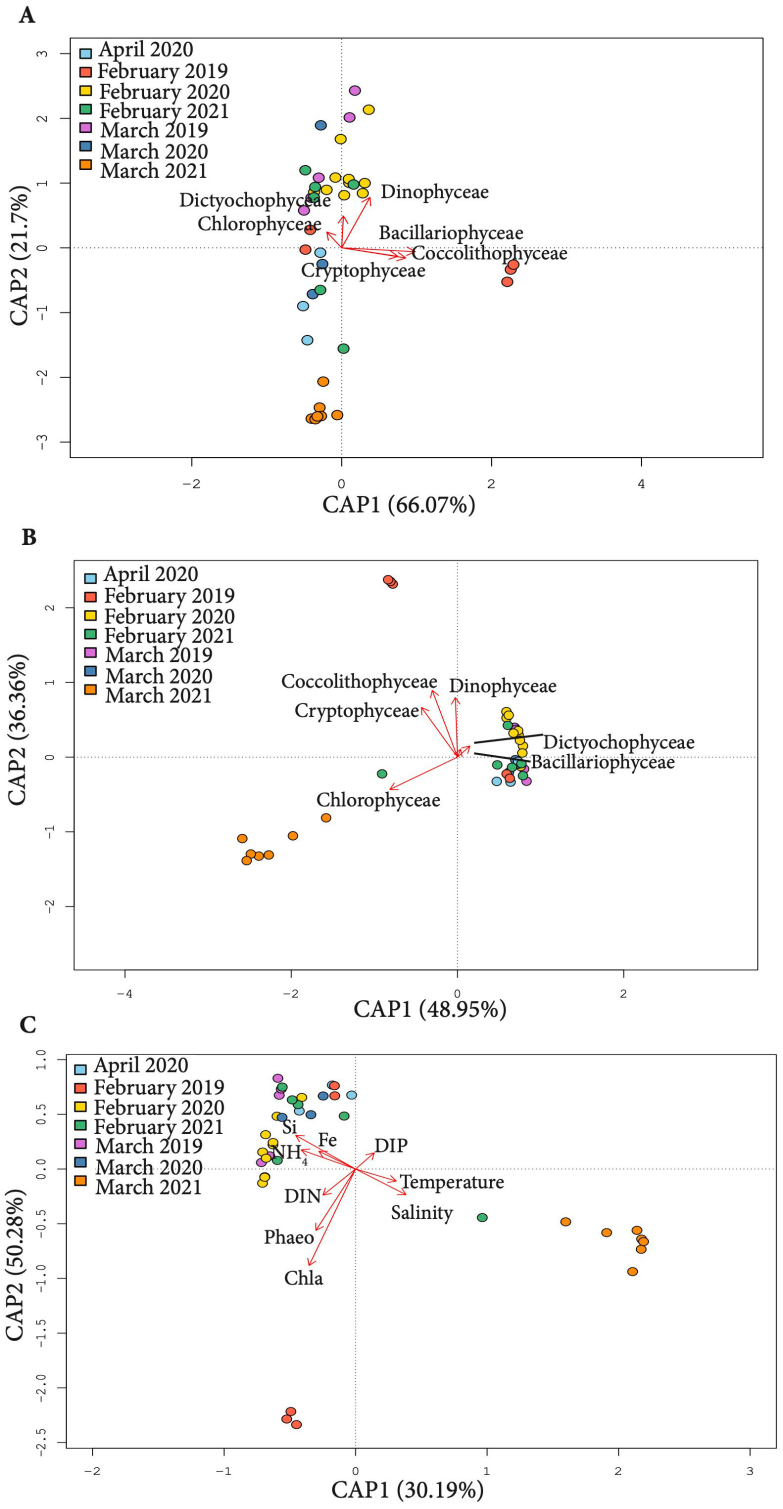
Canonical Analysis of Principal Components (CAP) of 16S rRNA profiles (OTUs) constrained by phytoplankton abundance **(A)**, biomass **(B)** and physico-chemical parameters **(C)** across the study period.

The samples clustered in three major groups under the influence of physico-chemical parameters and phytoplankton biomass/abundance (Figures 4A–C). Physicochemical parameters explained 50.28% and 30.19% variation along the first and the second ordination axes respectively (Figure 4C). Si, NH_4_ and Fe concentrations were driving factors shaping the bacterioplankton community structure in the majority of samples, whereas DIN, and phytoplankton pigments influenced February 2019 community. The variation in microbial community composition in March 2021 was primarily driven by the seawater temperature and salinity.

The relationship between phytoplankton and bacterioplankton dynamics varied depending on whether phytoplankton biomass or abundance was considered. *Dinophyceae, Dictiophyceae* and *Cholorophyceae* abundance explained some variation in microbial communities composition in March 2019, February 2020 and February 2021, whereas *Bacillariophyceae, Coccolithophyceae* and *Crytophyceae* abundance contributed to differentiation of February 2019 samples (Figure 4A).

Meanwhile, the biomass of *Coccolithophyceae, Cryptophyceae* and *Dinophyceae* contributed to shaping bacterioplankton structure in February 2019, while *Bacillariophyceae* had minor influence. *Chlorophyceae* biomass was the driving factor behind the differentiation of microbial communities in March 2021 (Figure 4B).

Taking into account the influence of biotic and abiotic factors on microbial community revealed by the CAP, Spearman correlation was performed to test for significant relationships between the dynamics of specific bacterioplankton taxonomic groups and environmental factors. A significant positive correlation (*p* < 0.05) was observed between the abundance of alphaproteobacterial clades - *Sulfitobacter, Yoonia*; and Chl *a* as well as with the biomass of *Bacillariophyceae, Dinophyceae* and *Coccolithophyceae* (Supplementary Figure 6A and Supplementary Table 4). Meanwhile, the strongest positive correlation was detected between the abundance of *Bacillariophyceae* and the abundance of *Sulfitobacter* (*R* = 0.59, *p* = 0.00007) and *Yoonia* (*R* = 0.53, *p* = 3.25e-009) (Supplementary Figure 6B and Supplementary Table 5). In contrast, most bacterioplankton taxa such as SAR11 clade Ia, *Colwellia, Planktomarina*, NS5, *Marinoscillum, Brevibacterium, Vicingus*, SAR92, *Polaromonas, Acinetobacter*, SUP05, *Ascidiaceihabitans* and *Sphingomonas*, showed a negative correlation with phytoplankton pigments (Supplementary Figures 6A, B). Additionally, a negative correlation was observed between the majority of bacterioplankton genera and phytoplankton biomass.

NH_4_ concentration correlated positively with *Dinophyceae* biomass and *Lentimonas* abundance, while showing a negative correlation with *Marinoscillum* and *Acinetobacter* abundance (Supplementary Figure 6A and Supplementary Table 4). Dissolved inorganic nitrogen concentration exhibited the positive correlations links with genera *Sulfitobacter, Yoonia, Marinoscillum* and *Vicingus* while *Lentimonas, Planktomarina*, SAR92 and *Ascidiaceihabitans* were negatively correlated (Supplementary Figures 6A, B). Dissolved inorganic phosphorus positively correlated with *Chlorophyceae* biomass, and the abundance of *Planktomarina*, SAR92, *Marinoscillum* and *Acinetobacter*, but negatively with *Coccolithophyceae* abundance and *Bacillariophyceae* biomass. A significant positive correlation was found between iron concentration, *Dictyochophyceae* biomass, and the abundance of *Colwellia, Polaromonas, Paraglaciecola* and *Marimonas*, while *Dinophyceae* and *Coccolithophyceae* biomass were negatively correlated with SAR11 clade Ia abundance (Supplementary Figures 6A, B).

Positive correlations were found between salinity and the abundance of bacterioplankton clades - *Lentimonas*, SAR11 clade Ia, SAR92, *Acinetobacter* and *Ulvibacter*, as well as phytoplankton biomass (*Chlorophyceae* and *Cryptophyceae*). In contrast, salinity showed a negative correlation with the abundance of *Polaribacter, Pseudoalteromonas, Candidatus* Aquiluna and *Dictyochophyceae* biomass (Supplementary Figure 6A). The abundance of *Polaromonas, Paraglaciecola* and *Marinomonas* was characterized by significant (*p* < 0.05) positive correlation with temperature, whereas *Dinophyceae* abundance was negatively correlated (Supplementary Figure 6FB).

More correlations were revealed when the interplay between phytoplankton and bacterioplankton was visualized separately for each sampling year. Distinct correlation patterns were observed between bacterioplankton genera and phytoplankton class abundances in 2019, 2020, and 2021, in line with differences in the taxonomic structure of the communities (Supplementary Figures 7A–C).

### 3.3 Functional dynamics of bacterioplankton communities

**Figure 5:**
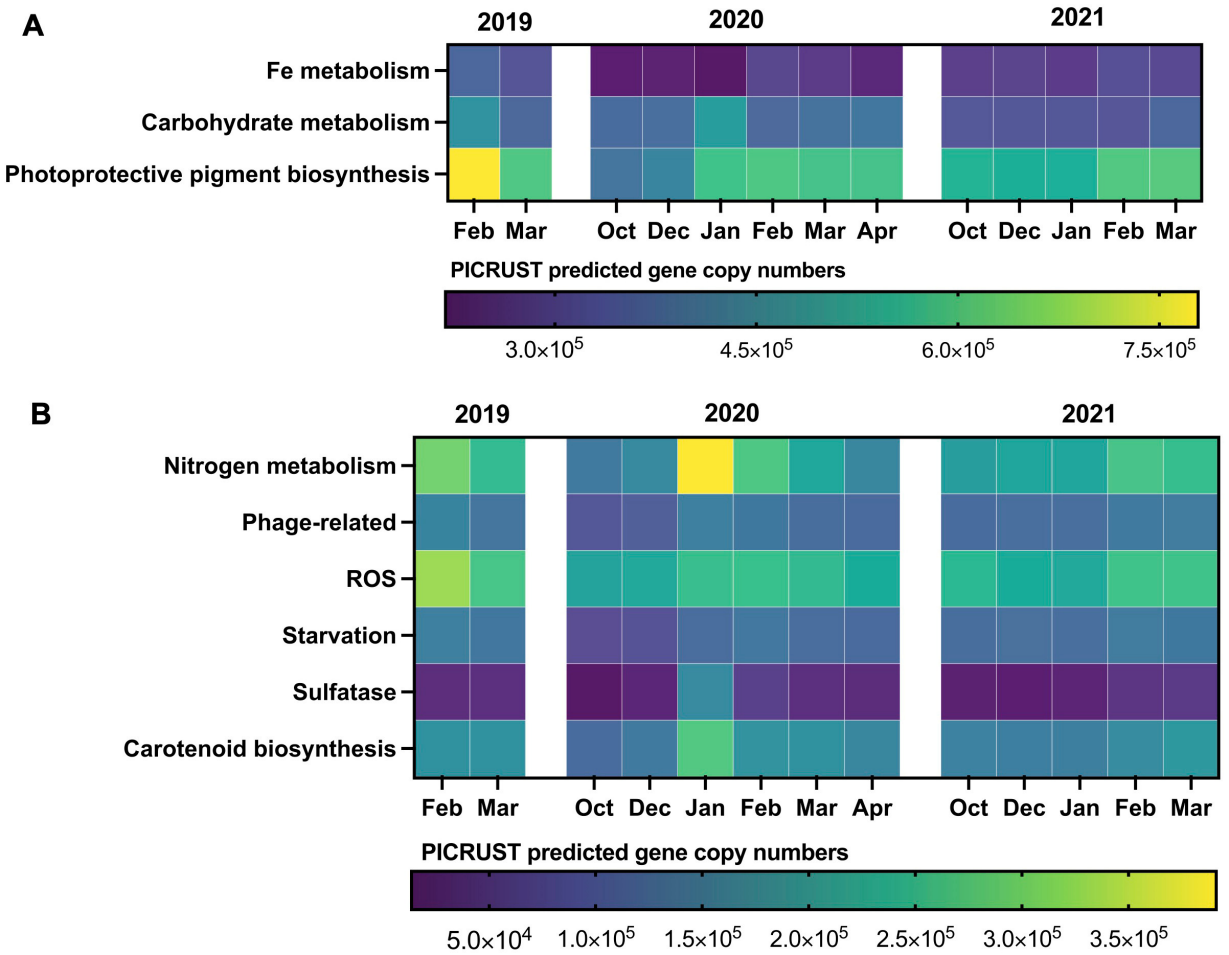
Interseasonal and interannual dynamics of PICRUST predicted functions (OTU-based) in bacterioplankton communities of Wilhelm Archipelago waters. **(A)** major functions, **(B)** minor functions.

PICRUSt (Phylogenetic Investigation of Communities by Reconstruction of Unobserved States) was used to indirectly predict the functional composition of the studied microbial community based on 16S rRNA gene sequencing data. Differential distribution of PICRUSt predicted microbial genes was observed throughout the sampling period, reflecting the interseasonal dynamics of environmental factors. Phosphorus metabolism genes were most abundant, with the highest predicted gene copy numbers of 1,378,853 and 1,249,057 in the bacterioplankton functional repertoire in February 2019 and January 2020, respectively. PICRUSt predicted photoprotective pigment biosynthesis genes showed constantly high levels, averaging for 574,148 copies, with a maximum peak in February 2019. Carbohydrate metabolism genes exhibited a mosaic distribution across seasons, reaching the maximum of 464,215 and 488,805 in February 2019 and January 2020, respectively (Figure 5A).

The interseasonal distribution of carotenoid biosynthesis genes, as predicted by PICRUSt, followed the dynamics of photoprotective pigment biosynthesis, with a few exceptions. The highest gene copy numbers were observed in the summer of 2019–2020 peaking at 278,681 in January 2020 (Figure 5B). Genes involved in mediating reactive oxygen species (ROS) distribution reached a maximum of 235,211 predicted copies in February 2019 and dropped to a minimum of 153,844 in October 2019. The PICRUSt predicted genes related to phage protection fluctuated similarly to starvation-related genes, with a minimum predicted of 85,877 and maximum of 153,351 copies (Figure 5B). Sulfatase genes peaked in January 2020 at 165,666 copies, maintaining consistently low levels for the remainder of the sampling period (Figure 5B). Nitrogen metabolism genes predicted by PICRUSt were most abundant in summers of 2019 and 2020, reaching 390,926 copies in January 2020. Genes involved in DMS and DMSP demethylation (*dmdA* and *dddL*) also showed peaks with 66,387 copies in February 2019 and 42,155 copies in January 2020 (Supplementary Table 6).

**Figure 6:**
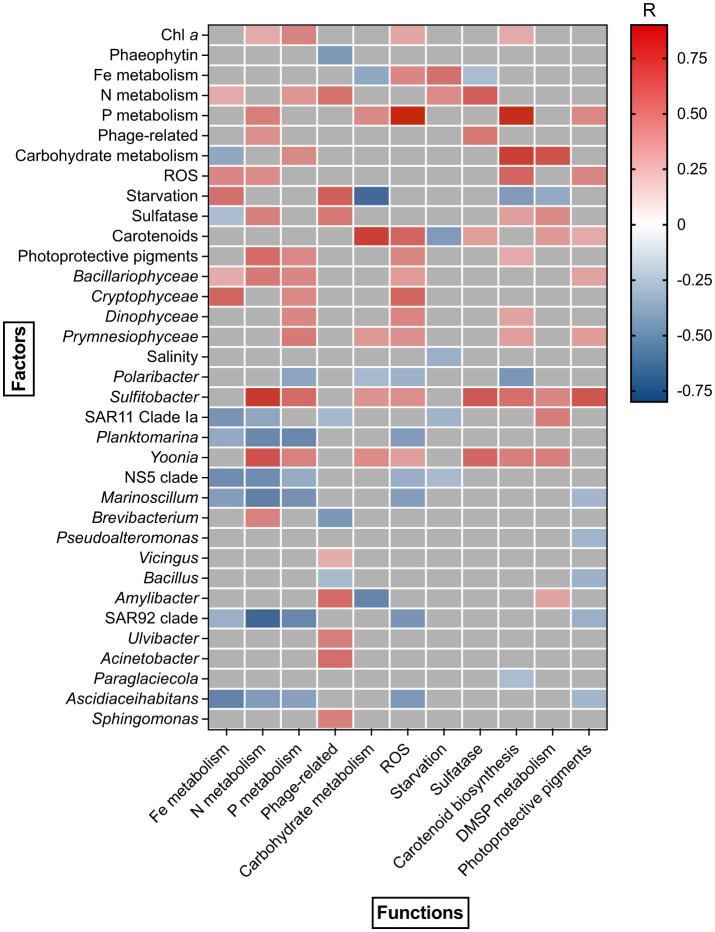
Spearman correlation between phytoplankton/bacterioplankton taxonomic structure and bacterioplankton functional composition during the study period.

A positive correlation was detected between Chl *a* concentration and PICRUSt predicted genes involved in N metabolism (*R* = 0.35, *p* < 0.05), P metabolism (*R* = 0.5, *p* < 0.05), ROS (*R* = 0.4, *p* < 0.05) and carotenoid biosynthesis (*R* = 0.35, *p* < 0.05) (Figure 6). Carbohydrate metabolism showed a positive correlation with phytoplankton (*Coccolithophyceae, R* = 0.42, *p* < 0.05) and bacterioplankton (*Sulfitobacter, R* = 0.45, *p* < 0.05, *Yoonia, R* = 0.48, *p* < 0.05) dynamics, as well as with carotenoid biosynthesis (*R* = 0.78, *p* < 0.05), P metabolism (*R* = 0.49, *p* < 0.05) and DMSP metabolism genes (*R* = 0.7, *p* < 0.05) (Figure 6). Similarly, N and DMSP metabolism, sulfatase, carotenoid and photoprotective pigments biosynthesis gene copy numbers correlated with the abundance of *Sulfitobacter* (*R* = 0.79, *R* = 0.5, *R* = 0.68, *R* = 0.59, *R* = 0.69, *p* < 0.05) and *Yoonia* (*R* = 0.7, *R* = 0.52, *R* = 0.62, *R* = 0.52, *R* = 0.63, *p* < 0.05). Most functional gene groups showed a positive correlation with *Bacillariophyceae* (e.g., *R* = 0.5, *p* < 0.05 for P metabolism) and *Cryptophyceae* (e.g., *R* = 0.62, *p* < 0.05 for Fe metabolism), while P metabolism, ROS and carotenoid biosynthesis were positively correlated with *Dinophyceae* (*R* = 0.5, *p* < 0.05, *R* = 0.5, *p* < 0.05, *R* = 0.39, *p* < 0.05).

A group of bacterioplankton genera showed negative correlations with nutrients metabolism. Particularly strong correlations were observed for Fe metabolism, *Ascidiaceihabitans* (*R* = −0.63, *p* < 0.05) and NS5 clade (*R* = −0.58, *p* < 0.05), as well as for P metabolism, *Planktomarina* (*R* = −0.6, *p* < 0.05), *Marinoscillum* (*R* = −0.56, *p* < 0.05) and SAR92 clade (*R* = −0.6, *p* < 0.05). N metabolism was characterized by a negative correlation with SAR11 clade Ia (*R* = −0.57, *p* < 0.05), *Planktomarina* (*R* = −0.6, *p* < 0.05), NS5 clade (*R* = −0.6, *p* < 0.05), *Marinoscillum* (*R* = −0.63, *p* < 0.05) and SAR92 clade (*R* = −0.76, *p* < 0.05) abundance (Figure 6). A strong negative correlation was observed between carbohydrate metabolism genes and *Amylibacter* (*R* = −0.61, *p* < 0.05) abundance, as well as between carotenoid biosynthesis genes and *Polaribacter* (*R* = −0.53, *p* < 0.05). No significant correlation was detected between bacterioplankton functional structure and environmental physical parameters, except for salinity, which showed a weak negative correlation with starvation-related genes (*R* = −0.4, *p* < 0.05) (Figure 6).

## 4 Discussion

Distinct interseasonal and interannual phytoplankton succession patterns, accompanied by corresponding bacterioplankton dynamics, were observed during the three consecutive sampling years in the Wilhelm Archipelago waters. The 2019 sampling year was characterized by a higher complexity in phytoplankton community structure, with two taxonomically distinct bloom events (dominated by *Coccolithophyceae* and *Bacillariophyceae*), whereas no mass-developments were observed in 2020 and 2021. This indicates a generally lower intensity of functional processes in the local phytoplankton community (Zotov et al., 2024), potentially resulting from differences in sea-ice season duration 2020 – 2021 compared to 2019 (Supplementary Figure 8). Indeed, a longer sea-ice season and later ice retreat are generally associated with higher phytoplankton biomass during subsequent spring and summer (Nardelli et al., 2023).

Accordingly, bacterioplankton seasonal differentiation was pronounced during summer–autumn transition of 2019 with two distinct clusters corresponding to the sequential development of phytoplankton taxa (Supplementary Figure 4A). Fewer microbial genera dominated in 2019, which may be linked to the selection of certain specialized bacterial taxa for phytoplankton substrate utilization (Seymour et al., 2017). Meanwhile, 2020 and 2021 were characterized by more heterogeneous bacterial community structure, as shown by diversity indices. A similar response was previously shown in both temperate (Costas-Selas et al., 2023) and polar waters (Richert et al., 2019; Cordone et al., 2022), where bacterioplankton diversity was higher in low-chlorophyll *a* scenario. A higher Shannon index observed during the 2020 and 2021 sampling years may have resulted from reduced competition with primary producers, allowing oligotrophic and mixotrophic groups to proliferate (Cordone et al., 2022). At the same time, microbial community phylogenetic diversity reflected by the PD index peaked late in February 2019, following the collapse of the *Coccolithophyceae* bloom, potentially indicating a response to increased availability of phytoplankton-derived organic matter (Buchan et al., 2014; Sidhu et al., 2023). In contrast, in the waters of the South Shetland Islands, a higher bacterioplankton Shannon index was associated with phytoplankton bloom compared to low-productivity periods (Fuentes et al., 2019). This discordance with our data may stem from the lower Chl *a* concentration observed in Southern Shetland waters compared to the values estimated for the Wilhelm Archipelago (2.5 and 7.0 mg^*^m^−3^ during bloom, respectively), implying potentially less competition from phytoplankton in the former.

A trend toward a decrease in the abundance of heterotrophic phytoplankton-associated bacteria and an increase in oligotrophic and chemolithoautotrophic taxa was detected from spring/summer to autumn, corresponding to the decline in phytoplankton abundance. *Polaribacter, Yoonia, Sulfitobacter, Amylibacter*, and SAR92 dominated during spring-summer, while the ratio of *Polaromonas* and *Paraglaciecola* increased in spring. In contrast, *Alphaproteobacterial* SAR11 clades Ia, II and III, along with *Gammaproteobacterial* SUP05, became dominant in autumn. This aligns with the general paradigm of marine productivity balance, which is driven by copiotrophic bacteria that respond quickly to increase in DOM and oligotrophic bacteria adapted to lower nutrient concentrations and utilization of low molecular weight substrates (Buchan et al., 2014; Sidhu et al., 2024). A similar trend and association with trophic conditions have been observed in multiple studies in Southern Ocean waters west of Palmer Station (Grzymski et al., 2012), the Ross Sea (Cordone et al., 2022), the Weddell Sea (Piontek et al., 2022), the Amundsen Sea Polynya (Richert et al., 2019) and the Arctic Ocean (Wietz et al., 2021).

The summer-autumn bacterioplankton community grouped in 3 clusters shaped by both biotic and abiotic factors. The microbial community in February 2019 was shaped by the concentration of phytoplankton pigments and the dynamics of *Bacillariophyceae, Coccolithophyceae*, and *Dinophyceae*, reflecting two successive, taxonomically distinct phytoplankton mass developments during that period. Positive correlation links were revealed between *Bacillariophyceae, Coccolithophyceae, Dinophyceaea*, and members of the bacterioplankton community – *Sulfitobacter* and *Yoonia*, which formed a distinct cluster based on their association with phytoplankton abundance and Chl *a* concentration. *Sulfitobacter* has previously been shown to benefit from sulfur compounds produced by phytoplankton (Amin et al., 2015), to respond positively to the addition of diatom-derived DOM in culture (Landa et al., 2014; Tisserand et al., 2020) and to constitute an essential part of the *Asterionellopsis glacialis* diatom microbiome (Isaac et al., 2024). Additionally, a stimulating effect of *Sulfitobacter* and *Yoonia* on the growth of diatom *Skeletonema marinoi* was discovered, highlighting their tight association (Johansson et al., 2019).

Likewise, *Cryptophyceae* abundance was positively correlated with *Vicingus* and *Polaribacter*. This correlation might demonstrate a true association, as both *Polaribacter* and *Vicingus* are known to be capable of alpha- and beta-glucan utilization (Grzymski et al., 2012; Wilkins et al., 2013), which are found in *Cryptophyceae*, similar to other phytoplankton taxa (Beidler et al., 2024). The Western Antarctic Peninsula is experiencing regional warming with reduced winter ice cover, leading to an increased role of low-biomass cryptophytes compared to diatoms (Brown et al., 2021). Thus, the low sea-ice winter of 2020–2021 (as indicated by lower sea-ice extent according to Palmer Station Antarctica LTER) may have resulted in an increased abundance of *Cryptophyceae*, which subsequently became a significant carbon source for *Polaribacter* during summer and autumn. Nevertheless, the absence of a clear correlation between the diatoms and *Polaribacter* is intriguing, as *Polaribacter* was previously identified as one of the major responders to diatom-dominated algal blooms (Piontek et al., 2022; Sidhu et al., 2023; West et al., 2024). One possible explanation is that the *Polaribacter* detected in our data may represent a clade adapted to polysaccharide degradation following algal bloom collapse, resulting in a time lag between these heterotrophic bacteria and diatoms (Avci et al., 2020).

Meanwhile, several negative correlations were identified between *Coccolithophyceae* and bacterial taxa such as *Ascidiaceihabitans, Ulvibacter, Lentimonas*, SUP05*, Planktomarina* and *Marinoscillum*. This may reflect competition between phyto- and bacterioplankton for nutrients that became limited during microalgae mass-development (Seymour et al., 2017; Costas-Selas et al., 2023). Indeed, multiple studies demonstrated that bacteria tend to outcompete phytoplankton for nitrogen and phosphorus in high-dissolved organic carbon availability (Buchan et al., 2014; Ratnarajah et al., 2021). Alternatively, the aforementioned bacterial taxa might have adapted to degrade low molecular weight organic matter, which becomes available later, after the bloom and initial breakdown of complex algal exudates (Teeling et al., 2016; Sidhu et al., 2024).

*Ulvibacter* was previously shown to respond during the later stages of bloom decline in Southern Ocean waters (Delmont et al., 2014; Liu et al., 2019) and to associate primarily with diatoms rather than haptophytes (West et al., 2024). In contrast to our findings, *Planktomarina* was shown to be abundant during phytoplankton blooms and to correlate with *Phaeocystis* in the Weddell Sea (Piontek et al., 2022), Southern Ocean waters off Kerguelen Island (West et al., 2024) and waters of the Australian continental shelf (O'Brien et al., 2022). However, the absence of polysaccharide degradation genes in the RCA cluster suggests that members of *Planktomarina* may rely on other low-molecular weight algal metabolites such as DMSP, DMS and taurine (Sidhu et al., 2024).

The dynamics of the bacterioplankton community functional structure, predicted using PICRUSt2 pipeline, aligned with phytoplankton development and environmental physical/chemical parameters. Specifically, a higher copy number of predicted carbohydrate degradation and sulfatase genes, which encode enzymes for the breakdown of algal sulfated polysaccharides, was observed in summer when diatoms and haptophytes proliferated. Similar patterns were reported during diatom blooms in the North Sea (Orellana et al., 2022; Sidhu et al., 2023), the Iberian Peninsula coastal upwelling zone (Pontiller et al., 2022), Fram Strait (Priest et al., 2023), the Weddell Sea (Piontek et al., 2024), and other marine ecosystems globally.

Genes predicted to enable the utilization of a range of carbohydrates peaked at different seasons, suggesting a microbial community response to the availability of diverse substrates. For example, genes coding for the utilization and transport of mannose, alpha-glucan, beta-galactose, alpha-galactose and glucose were more abundant in summer 2019 following the sequential mass development of haptophytes and diatoms. Spring 2019 was characterized by a marked dominance of predicted beta-galactosidase and fucose metabolism genes, with a secondary peak observed in summer 2019–2020.

DMSP demethylation genes peaked in summers of 2019 and 2020, coinciding with ice retreat (Supplementary Table 6) and the mass-development of *Phaeocystis antarctica* (*Coccolithophyceea*). High DMSP concentrations have been estimated in Antarctic sea ice (Asher et al., 2011) and incubation experiments with Southern Ocean plankton communities have shown that increased irradiance during meltdown events triggers DMSP production as an adaptive response to light-induced oxidative stress (Stefels and van Leeuwe, 1998; Sunda et al., 2002; Vance et al., 2013). Consequently, bacterial demethylation genes become more abundant when DMSP is released into the marine environment from melting ice and decaying phytoplankton cells. PICRUSt-predicted carbohydrate degradation and sulfatase genes correlated with *Sulfitobacter* and *Yoonia*, while DMSP degradation was also associated with SAR11 clade Ia. The results corroborated with studies that showed the utilization of sulfur-containing algal metabolites by SAR11 clade Ia (Tripp et al., 2008; Sidhu et al., 2024).

An increase of predicted carotenoid and photoprotective pigments synthesis genes was observed during periods of lower salinity and higher temperatures, indicating ice melt, and presumably higher light availability. The beneficial effect of carotenoid pigmentation on the survival of Antarctic bacterioplankton was demonstrated in an incubation study under freeze-thaw and solar radiation exposure (Dieser et al., 2010). Genes responsible for defense against reactive oxygen species (ROS) followed a similar trend, with higher copy numbers observed during periods of more intensive UV light exposure in summer. The release of H_2_O_2_ by diatoms, known to occur in Antarctica after rapid ice retreat (Morris et al., 2022), and the increased oxygen solubility at freezing temperatures (Tribelli and López, 2018) may have additionally contributed to the high activity of ROS-defense mechanisms in bacterioplankton. Similar to our findings, a significant increase in the abundance of superoxide dismutase and catalase genes was detected in Antarctic Peninsula waters during summer compared to dark winter period (Grzymski et al., 2012).

Predicted bacterial nutrient (nitrogen, phosphorus and iron) metabolism genes were more abundant during the Antarctic summer, coinciding with presumably higher ecosystem productivity. Nitrogen metabolism was predominantly associated with denitrification, which may be linked to particles formed by decaying phytoplankton cells (Ciccarese et al., 2023). Additionally, PICRUSt-predicted genes related to uric acid degradation outnumbered other nitrogen metabolism pathways, indicating an ornithogenic impact on the marine ecosystem (Parnikoza et al., 2018; Prekrasna-Kviatkovska et al., 2024).

Correlation was observed between temperature/salinity and bacterioplankton dynamics. This may result from the initial influence of the physical environment on phytoplankton, which subsequently drives microbial community succession (Bozzato et al., 2019; Hernando et al., 2020). A strong negative correlation between DIN concentration and seawater temperature was detected, which could be attributed to the inflow of nitrogen-rich nutrients from adjacent penguin colonies during melt events, associated with a drop in water temperature (Parnikoza et al., 2018; Prekrasna-Kviatkovska et al., 2024).

## 5 Conclusion

Microbial community taxonomic and functional structure was studied during the three consecutive years in the waters of Western Antarctic Peninsula, which represents a highly dynamic ecosystem with contrasting seasonal conditions.

Bacterioplankton composition shifted seasonally, with heterotrophic phytoplankton-associated bacteria dominating in spring/summer. In contrast, oligotrophic and chemolithoautotrophic taxa increased in autumn, coinciding with phytoplankton decline. Positive correlations were revealed between specific bacterioplankton genera and phytoplankton *Bacillariophyceae, Coccolithophyceae, Dinophyceaea* abundance, while higher predicted copy number of carbohydrate degradation and sulfatase genes observed during the periods of haptophyte and diatom development highlighted the tight association between Antarctic heterotrophic bacteria and microalgae.

Meanwhile, an increase in PICRUST predicted genes encoding carotenoid and photoprotective pigments synthesis coincided with the periods of lower salinity and higher temperatures, indicating a putative response to increased light availability during the ice melt. Additionally, a prevalence of PICRUSt-predicted uric acid degradation genes over other nitrogen metabolism pathways was detected, which signals an ornithogenic impact from the expanding penguin colony on the marine ecosystem.

These findings underscore the intricate interplay between bacterioplankton and phytoplankton in shaping microbial community dynamics and biogeochemical cycling in the rapidly changing Western Antarctic Peninsula ecosystem.

## Data Availability

The datasets presented in this study can be found in online repositories. The names of the repository/repositories and accession number(s) can be found in the article/Supplementary material.
