# Supplementary material for: Dynamics of microbial communities in Western Antarctic Peninsula waters shaped primarily by the biological interactions
Source: Front Microbiol. 2025 Jun 25;16:1591986. doi: 10.3389/fmicb.2025.1591986 (PMC12239745; doi:10.3389/fmicb.2025.1591986)
Supplement: Supplementary file 1 [file Supplementary_file_1.docx]

***Supplementary Material***

**Dynamics of microbial communities in Western Antarctic Peninsula waters shaped primarily by the biological interactions**

**Pavlovska M^*1^, Zotov A.^2^, Prekrasna-Kviatkovska Ye.^1^, Sidhu C.^3^, Dzhulai A.^4^, Dzyndra M.^1^, Dykyi E.^1^**

^1^State Institution National Antarctic Scientific Center, 16 Taras Shevchenko Blvd., 01601, Kyiv, Ukraine

^2^State Institution Institute of Marine Biology of the NAS of Ukraine, Odesa, Ukraine

^3^Max Planck Institute for Marine Microbiology, Celsiusstraße 1, 28359, Bremen, Germany

^4^University of Rhode Island, Graduate School of Oceanography, Narragansett, Rhode Island, 02882, USA

*** Correspondence:**Dr. Mariia Pavlovska
[mawwwa88@gmail.com](mailto:mawwwa88@gmail.com)

[mariia.pavlovska@uac.gov.ua](mailto:mariia.pavlovska@uac.gov.ua)

# Supplementary Data

# 1.1 Correlation patterns between phytoplankton and bacterioplankton abundance during each sampling year

*Coccolithophyceae* showed multiple strong negative correlations (R = -0.7 to -0.9, p < 0.05) with bacteria such as *Ascidiaceihabitans, Ulvibacter, Lentimonas,* SUP05*, Planktomarina* and *Marinoscillum* in 2019 (Figure S6A). *Bacillariophyceae* correlated negatively with *Amylibacter* and *Acinetobacter*, but positively with *Sulfitobacter* and *Yoonia*. Positive correlations were also found between *Chlorophyceae*, *Ulvibacter*, *Marimonas*, *Pseudoalteromonas* and *Vicingus*, as well as between *Dictyochophyceae* and *Sphingomonas.* Meanwhile, *Dinophyceae* correlated negatively with *Acinetobacter* and *Polaribacter*, and *Chlorophyceae* – with SAR11 clade Ia (Figure S6A). The only significant correlations detected between phytoplankton and bacterioplankton in 2020 were between *Bacillariophyceae* with SAR11 clade Ia, *Planktomarina*, NS5, SAR92 and *Ascidiaceihabitans* (all negative), and with *Amylibacter* (positive)*.* The correlations were generally weaker than those observed in 2019 (R = -0.6 to -0.7 and R = 0.5) (Figure S6B).

Multiple correlations were found between members of the phytoplankton and bacterioplankton communities in 2021. *Dictiochophyceae* was positively associated with *Ascidiaceihabitans* and SAR11 clade Ia but negatively with *Vicingus*, *Colwelia*, *Marimonas* and *Pseudoalteromonas*. *Dinophyceae* abundance correlated positively with *Ulvibacter* and *Ascidiaceihabitans*, but negatively with *Bacillus*, *Polaribacter*, *Paraglaciecola*, *Brevibacterium* and *Colwellia*. Positive association was detected for *Cryptophyceae* with *Vicingus* and *Polaribacter*, while negative correlations were observed with *Lentimonas*. Negative correlations were also found between *Bacillariophyceae*, *Paraglaciecola* and *Polaribacter*, as well as between *Chlorophyceae* and *Amylibacter* (Figure S6C)*.* The correlation coefficients were lower than in 2019 but higher than in 2020 (R = -0.5 to -0.5 and R = 0.5 to 0.8).

**1.2 Supplementary discussion of functional traits**

Genes for the utilization of fucose, mannose and alpha-glucan were also abundant during early autumn in 2019, 2020 and 2021 (Table S6). Although the absence of data on spring phytoplankton dynamics complicates the interpretation of bacterial data, the observed seasonal coherence in microbial community taxonomy and functions indicates a strong response to microalgal polysaccharides.

An increase in PICRUSt-predicted polysaccharide degradation genes was accompanied by a higher copy number of starvation-related genes, aligning with the competition for essential nutrients during phytoplankton mass-development (Ratnarajah et al. 2021, Costas-Selas et al. 2024). Mannose and galactose are known to be among the most abundant water-soluble carbohydrates in *Phaeocystis* (Janse et al., 1996), while alpha-glucan serves as a storage polysaccharide in bacteria and can be recycled from dead microbial cells (Deppeler et al., 2020, Biggs et al., 2021, Beilder et al., 2024).

Genes predicted to code for phage defense mechanisms were generally abundant throughout the sampling period, with higher estimates during phytoplankton mass development. This aligns with previous findings on phage proliferation during spring and summer months in Southern Ocean waters, which occur alongside increased bacterioplankton abundance and nutrient availability (Evans et al., 2017). Conversely, viral pressure on bacterioplankton persists during low Chl *a* periods, with distinct phage groups specializing in infecting bacteria rather than algae (Alarcón-Schumacher et al., 2019, Lopez-Simon et al., 2023).

Weak correlations were observed between hydrochemical parameters and plankton development, except for dissolved inorganic nitrogen. This suggest that no strict resource limitation occurred during the sampling campaign, and nutrient concentrations did not play a major role in shaping the communities (Annett et al., 2017, Sherrell et al., 2018, Calvalho et al., 2020). Additionally, the lack of correlations with hydrochemical parameters may be due to the fact that nutrient concentrations measured during phytoplankton development reflect the remnants of what was consumed by marine organisms, rather than the conditions that initially facilitated the increase in biomass and abundance. Collecting additional data across different seasons could reveal more comprehensive correlation patterns.

The negative correlation of SUP05 with *Coccolithophyceae* aligned with their preference for low-Chl *a* waters in the Weddell Sea (Piontek et al., 2022) and their distribution during Antarctic winter (Grzymski et al., 2012).

# Supplementary Figures and Tables

## Supplementary Figures


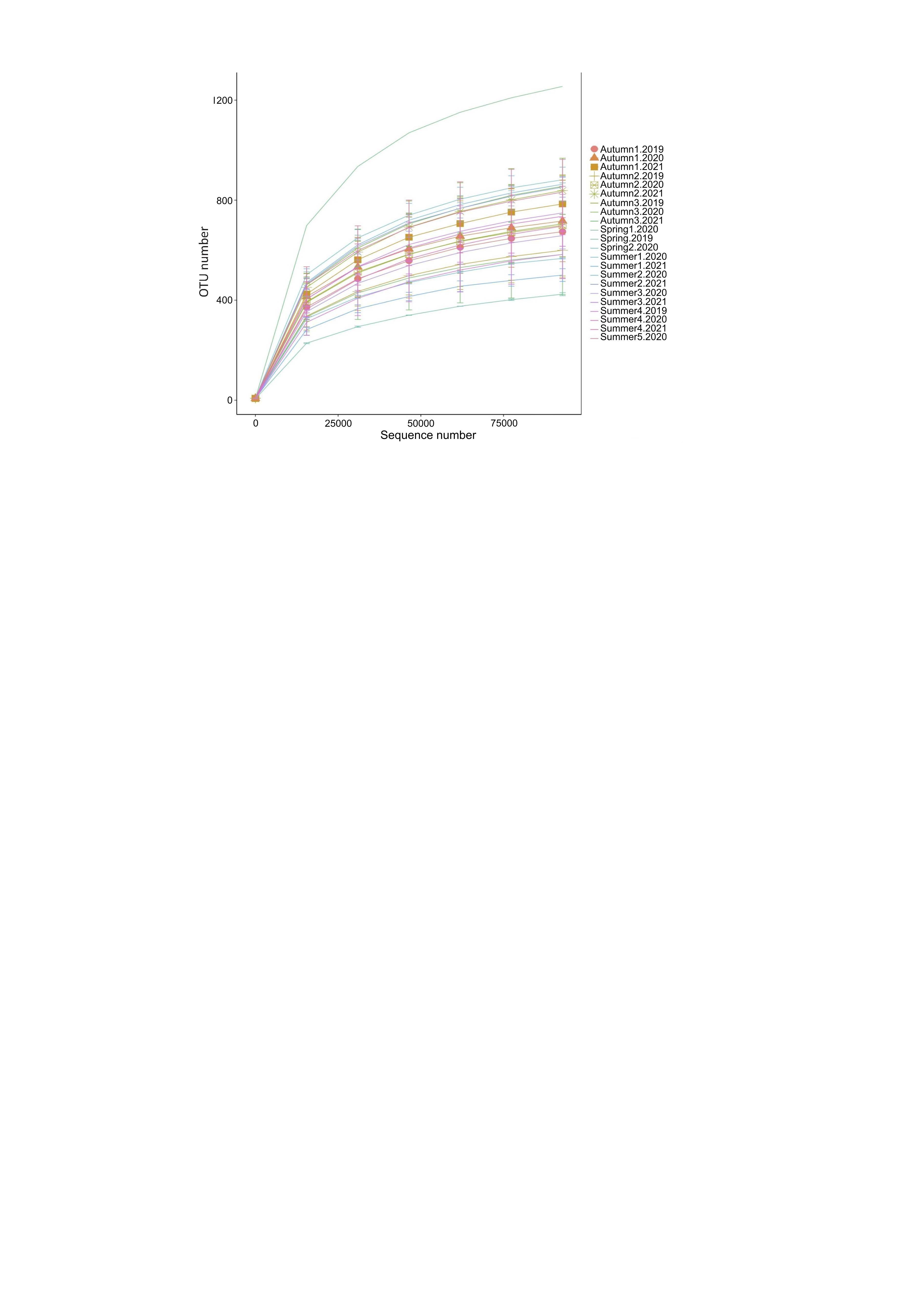


**Supplementary Figure S1**. Rarefaction curves for each sample processed


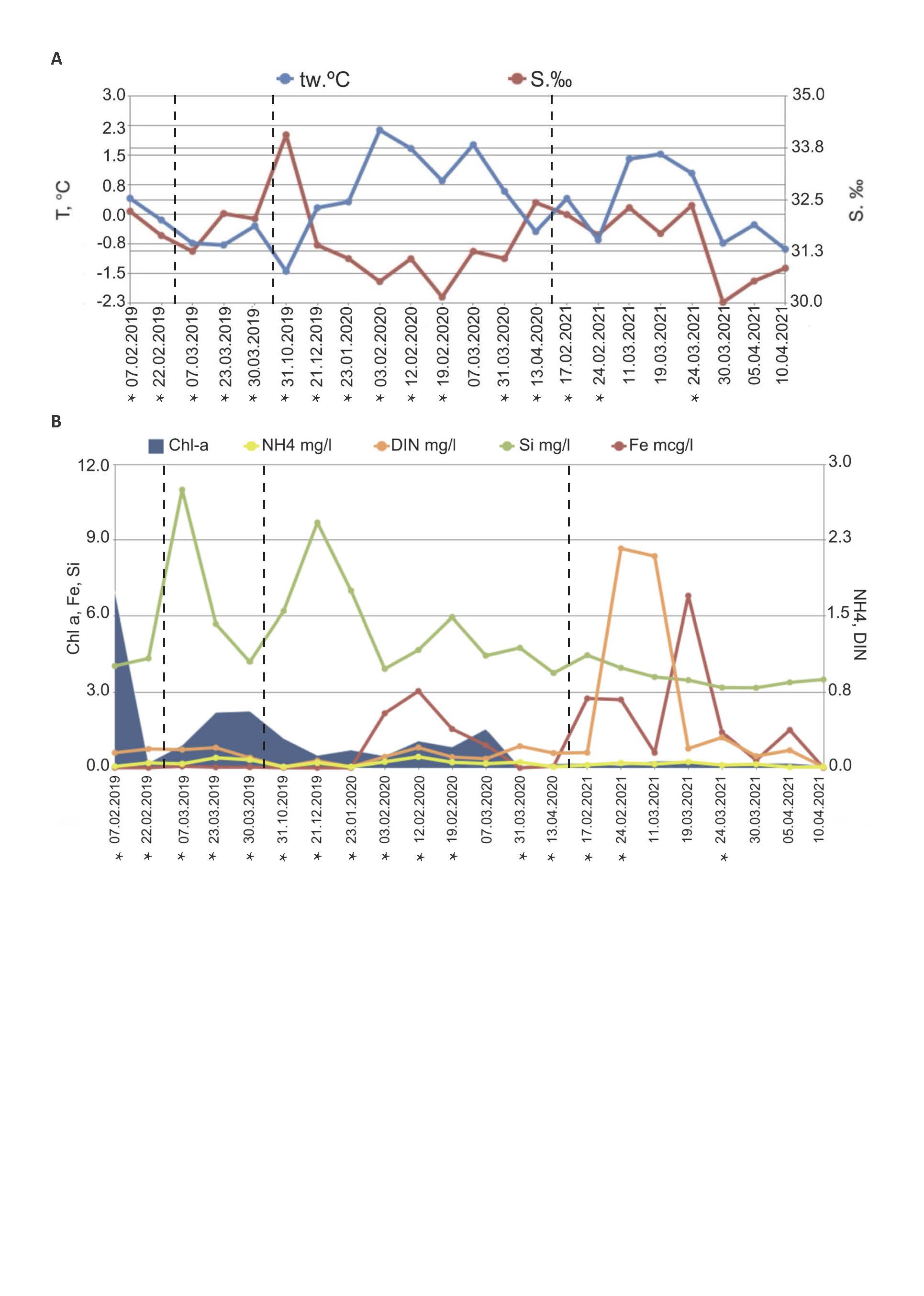


**Figure S2**. Dynamics of physico-chemical parameters during the survey period: (A) temperature and salinity, (B) Chl *a*, ammonium (NH_4_), dissolved inorganic nitrogen (DIN), silica (Si), iron (Fe). The sampling dates for bacterioplankton are indicated with asterisk, while the dotted vertical line shows the start of the corresponding phytoplankton-growing season


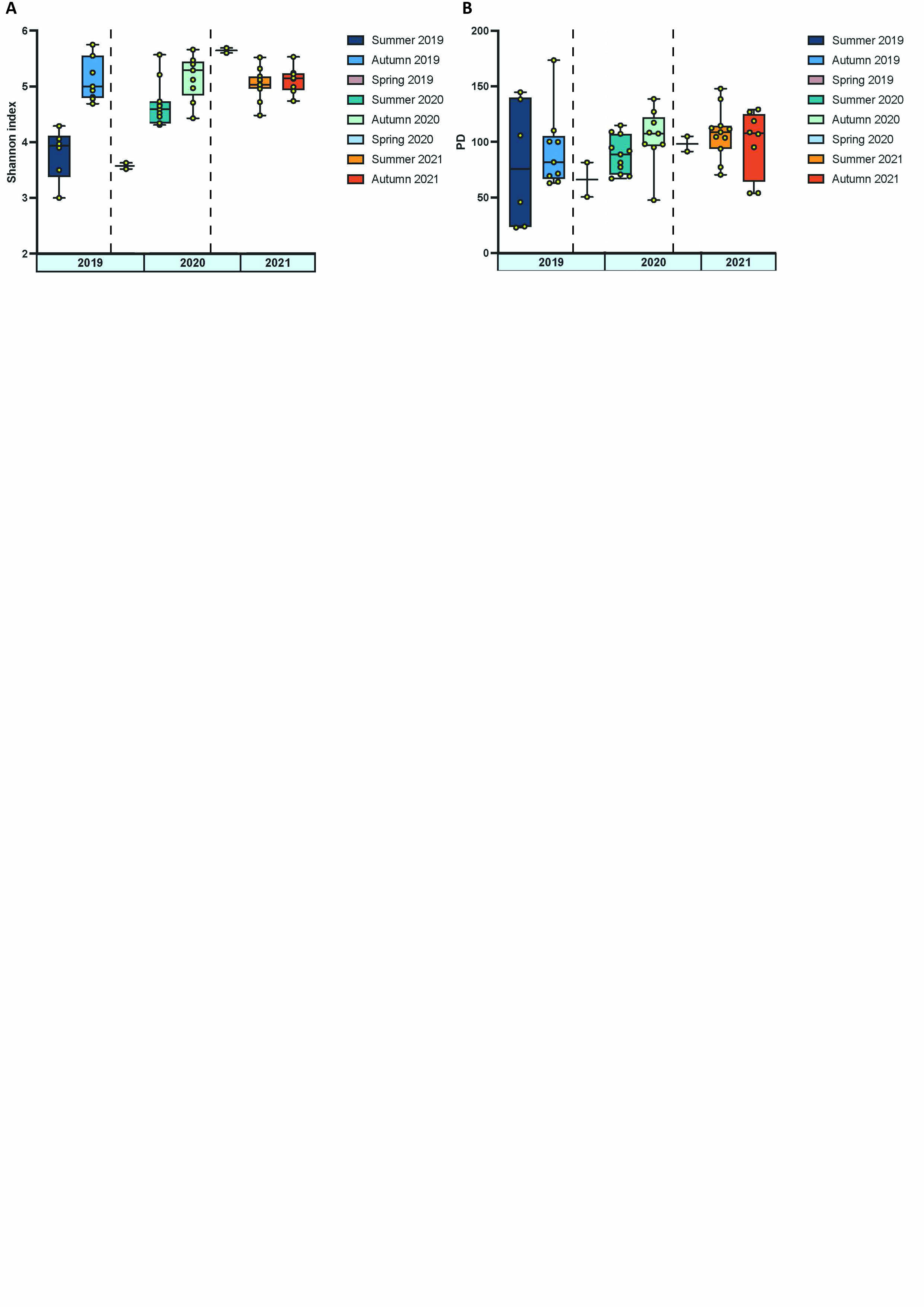


**Figure S3.** Bacterioplankton diversity dynamics during the sampling period.: (A) Shannon Index, (B) PD (Phylogenetic diversity index). The dotted vertical line shows the start of the corresponding phytoplankton growing season.


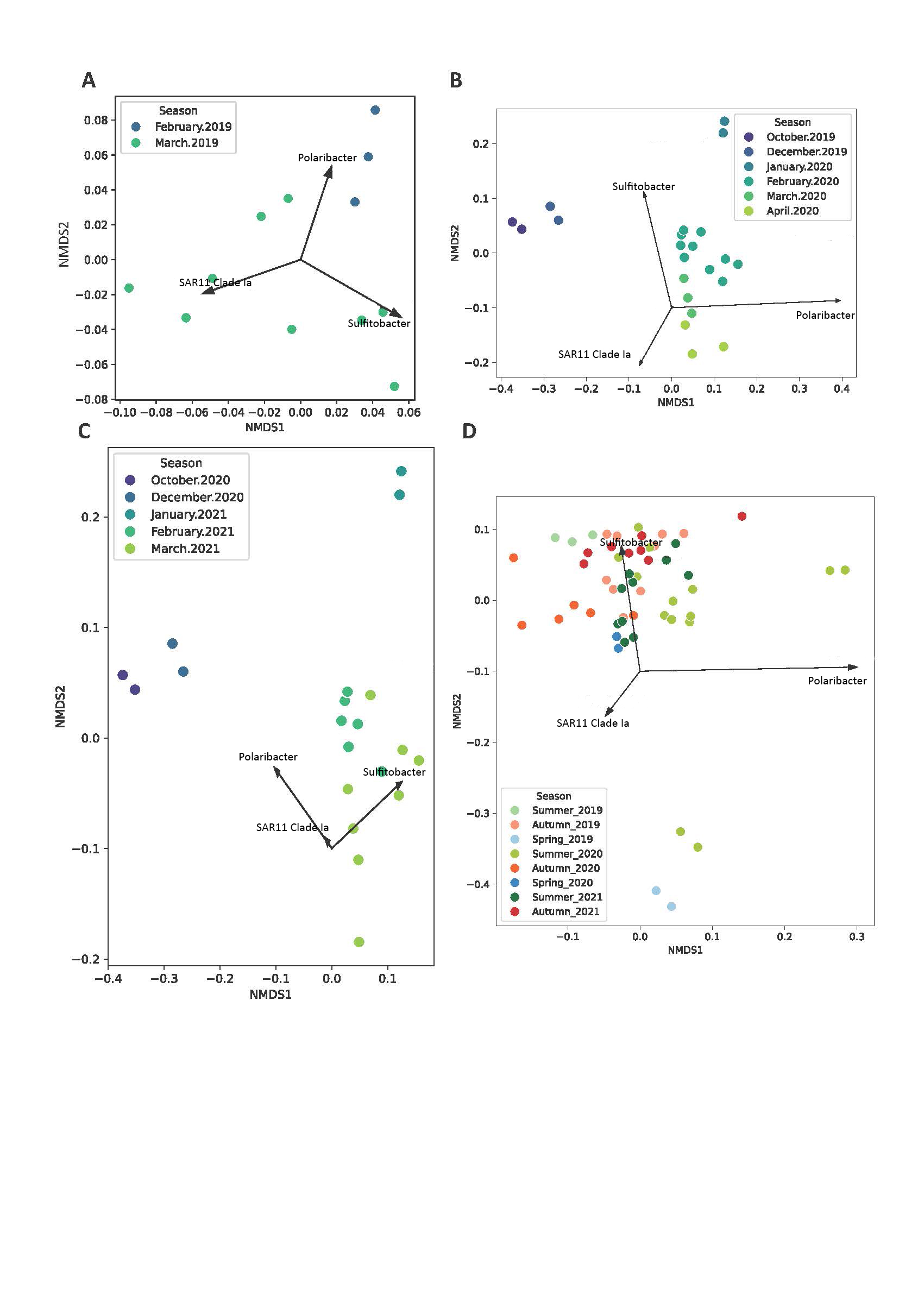


**Figure S4.** Interseasonal bacterioplankton differentiation represented by NMDS analysis: (A) 2019, (B) 2020, (C) 2021.


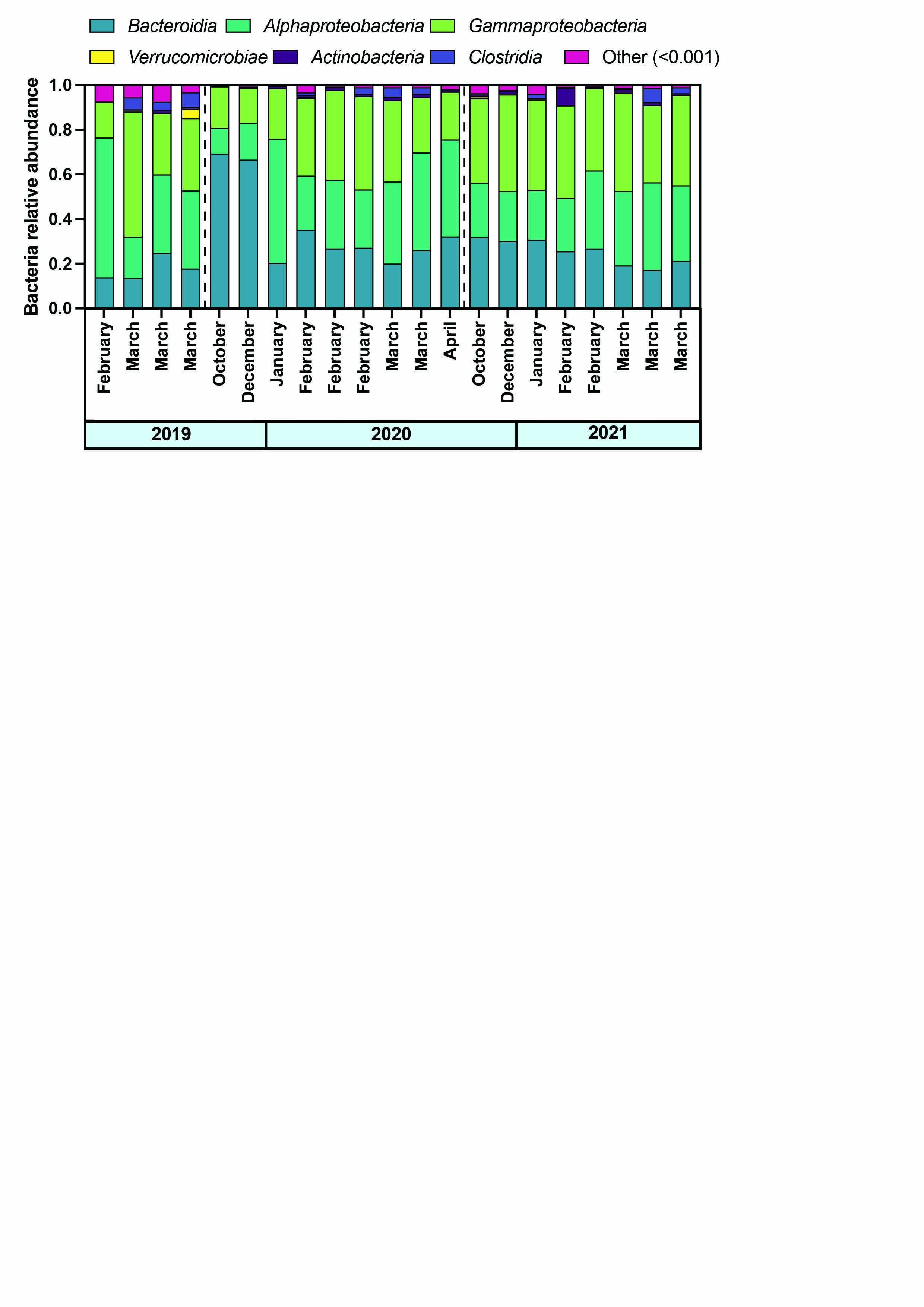


**Figure S5**. Bacterioplankton taxonomic structure at the class level. The dotted vertical line shows the start of the corresponding phytoplankton growing season


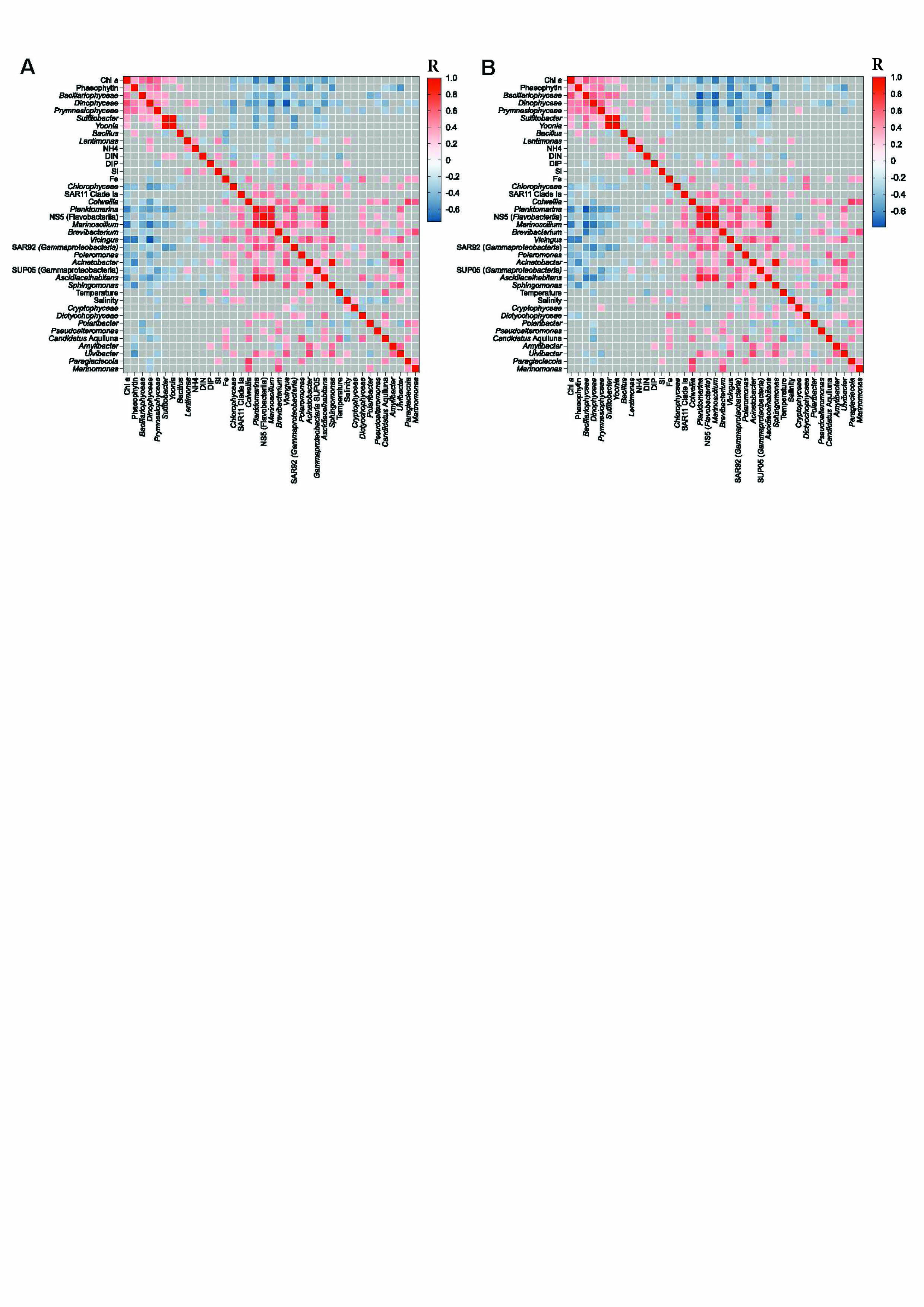


**Figure S6**. Spearman correlation between environmental physico-chemical parameters, bacterioplankton taxonomic structure and phytoplankton: A) biomass, B) abundance

**A**


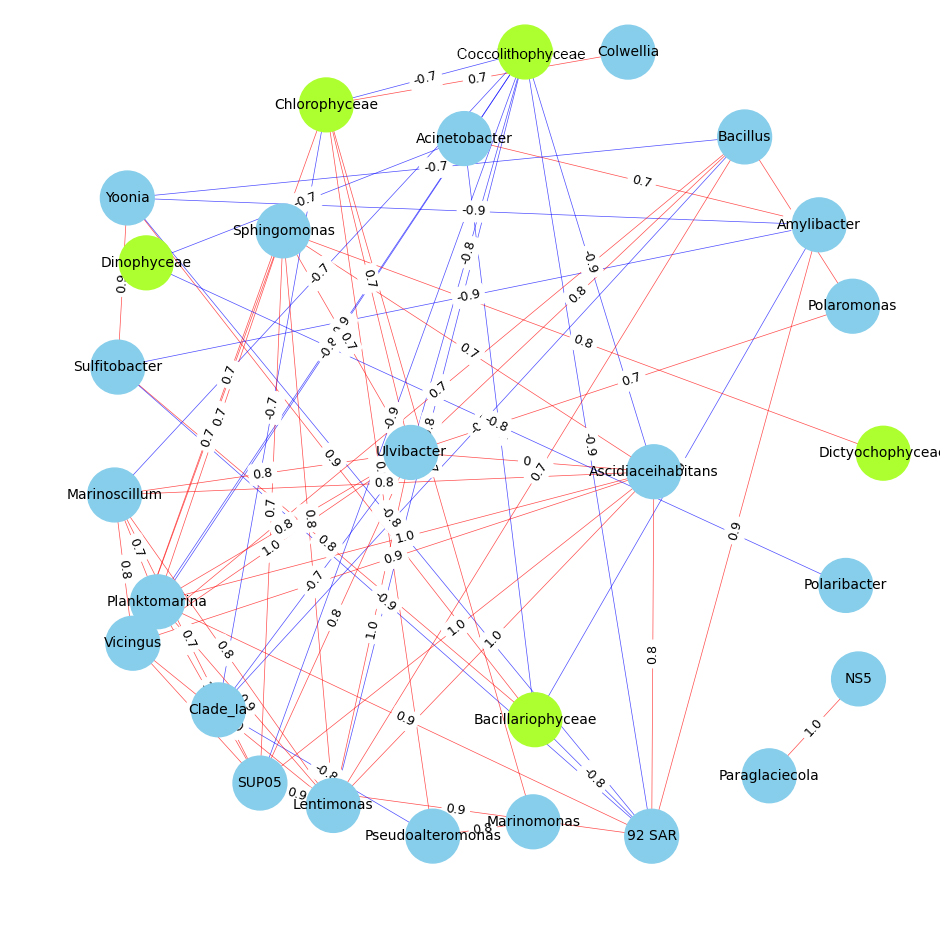


**B**

**
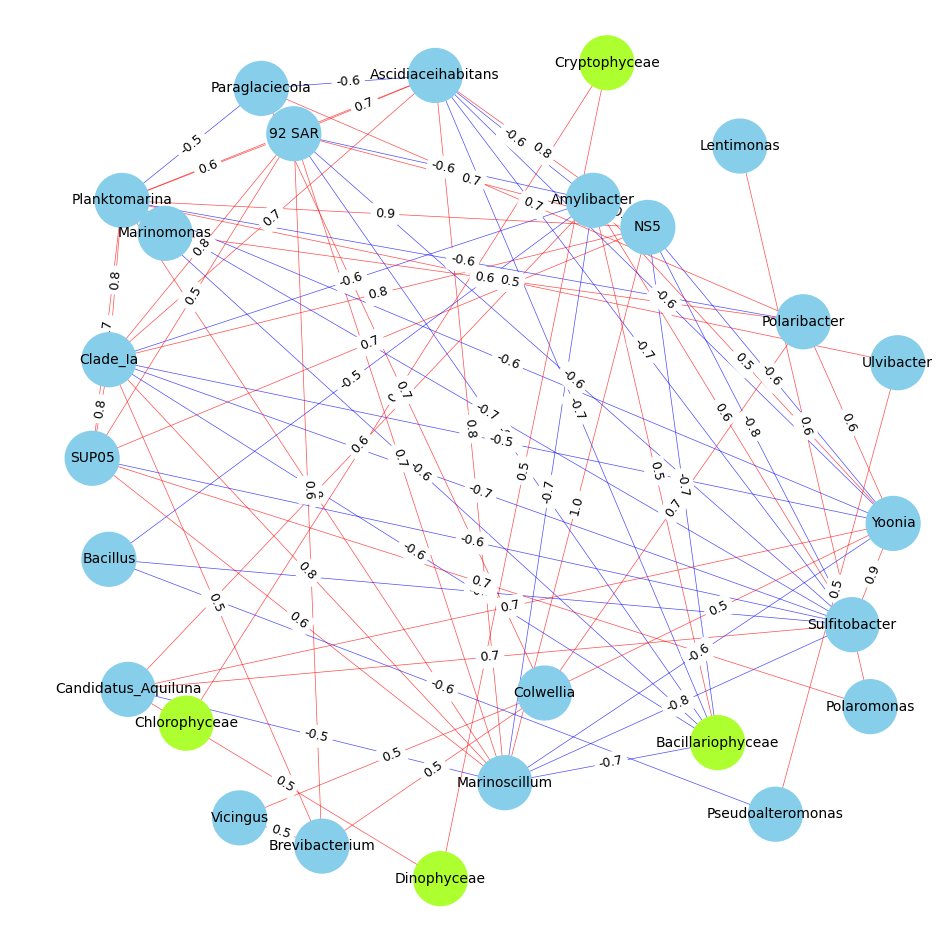
**

**C**

**
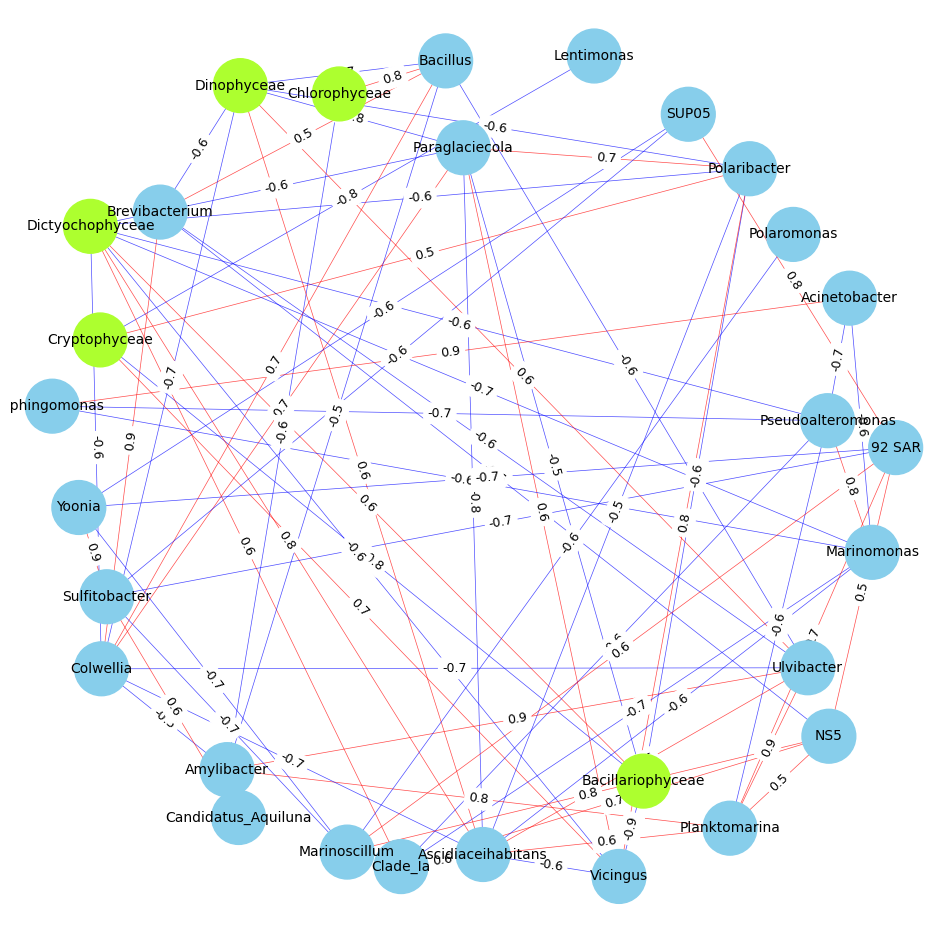
**

**Figure S7**. Correlation networks between phytoplankton and bacterioplankton abundance in : (A) 2019, (B) 2020 and (C) 2021. The numbers denote Spearman correlation coefficient – R. Positive correlation is depicted by the red lines, whereas negative ­– by the blue lines. Phytoplankton taxa are marked with the green circles and bacterioplankton taxa – with the light-blue circles


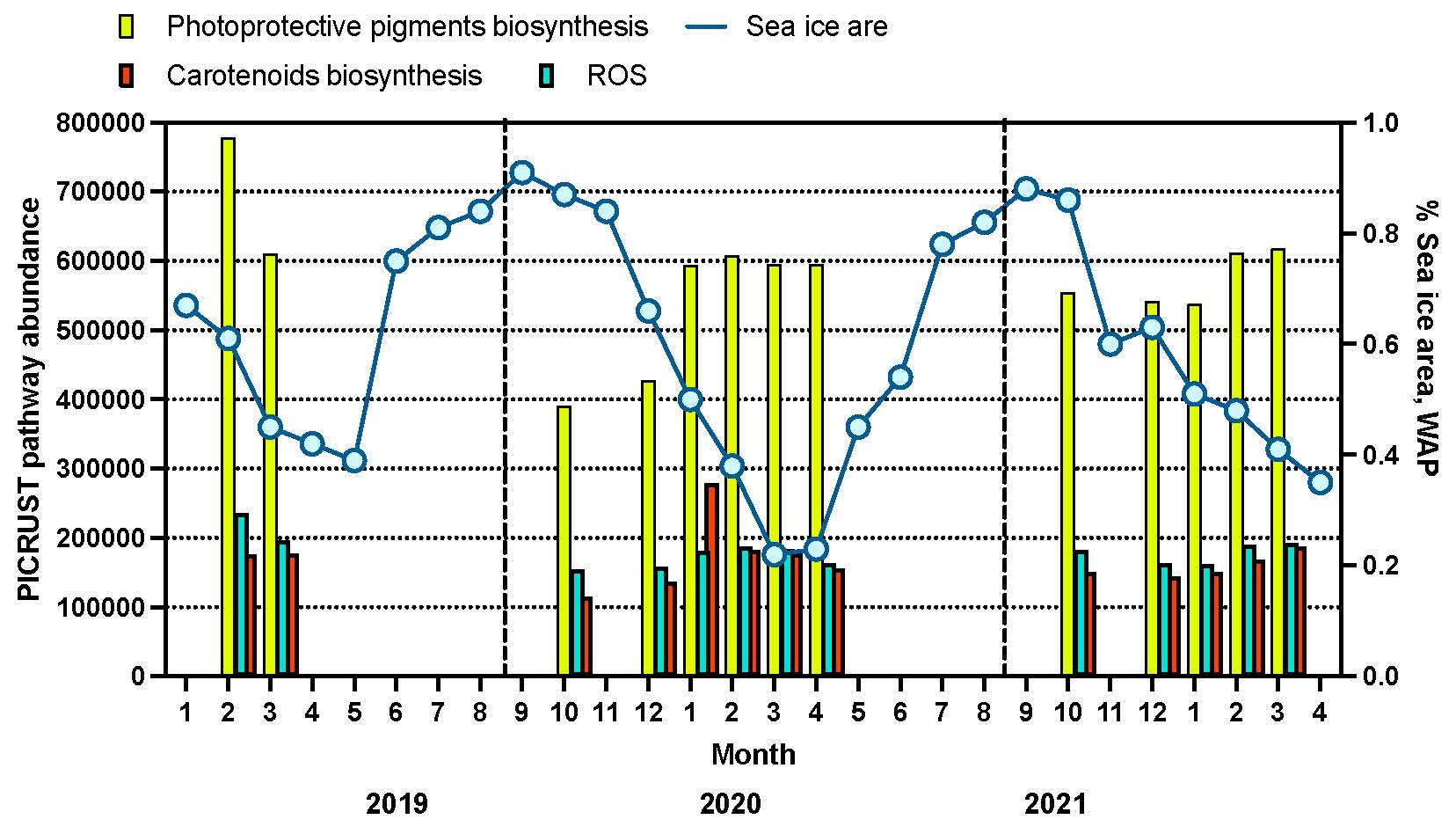


**Figure S8.** Dynamics of predicted ROS defence, photoprotection and carotenoid biosynthesis genes in relation to the ice area during the study period

## Supplementary Tables

**Table S1.** Location of the sampling stations in the waters of Wilhelm Archipelago (Antarctica)

| **Station** | **Location** | **Depth** |
| --- | --- | --- |
| 1 | 65°15'45"S 64°11'28"W | 1 m |
| 2 | 65°15'07"S 64°11'33"W | 1 m |
| 3 | 65°14'48"S 64°14'32"W | 1 m |
| 4 | 65°14'40"S 64°15'10"W | 1 m |
| 5 | 65°14'48"S 64°15'16"W | 1 m |

**Table S2.** Sequencing statistics

| Date | Category | Raw PE(#) | Combined | Qualified | Nochime | Base(nt) | AvgLen(nt) | Q20 | Q30 | GC% | Effective% |
| --- | --- | --- | --- | --- | --- | --- | --- | --- | --- | --- | --- |
| 07.02.19 | Summer.2019 | 135 208 | 121 562 | 118 721 | 90 573 | 37 848786 | 418 | 97.91 | 93.49 | 49.93 | 66.99 |
| 07.02.19 | Summer.2019 | 141 369 | 122 436 | 119 327 | 93 85 | 39 023909 | 416 | 97.74 | 93.16 | 50.57 | 66.39 |
| 07.02.19 | Summer.2019 | 140 995 | 129 998 | 127 82 | 94 984 | 39 546409 | 416 | 98.06 | 93.84 | 49.99 | 67.37 |
| 22.02.19 | Summer.2019 | 182 439 | 159 331 | 156 534 | 120 329 | 50 331982 | 418 | 97.92 | 93.36 | 50.66 | 65.96 |
| 22.02.19 | Summer.2019 | 185 134 | 162 012 | 159 307 | 132 286 | 54 441235 | 412 | 98.02 | 93.63 | 50.02 | 71.45 |
| 22.02.19 | Summer.2019 | 173 979 | 151 965 | 149 531 | 123 131 | 50 777918 | 412 | 97.98 | 93.55 | 50.46 | 70.77 |
| 22.02.19 | Summer.2019 | 170 924 | 149 858 | 147 081 | 117 746 | 49 014409 | 416 | 97.91 | 93.36 | 50.12 | 68.89 |
| 22.02.19 | Summer.2020 | 175 289 | 153 274 | 150 634 | 126 218 | 52 134419 | 413 | 97.89 | 93.27 | 49.57 | 72.01 |
| 07.03.19 | Autumn.2019 | 176 43 | 155 456 | 152 852 | 111 557 | 46 520245 | 417 | 97.92 | 93.40 | 50.76 | 63.23 |
| 07.03.19 | Autumn.2019 | 183 23 | 162 331 | 159 886 | 116 829 | 48 336898 | 414 | 97.92 | 93.34 | 50.42 | 63.76 |
| 07.03.19 | Autumn.2019 | 183 223 | 162 031 | 159 271 | 115 782 | 48 203306 | 416 | 97.96 | 93.42 | 50.60 | 63.19 |
| 07.03.19 | Autumn.2019 | 175 181 | 154 523 | 152 017 | 116 863 | 48 537363 | 415 | 97.96 | 93.45 | 50.88 | 66.71 |
| 07.03.19 | Autumn.2019 | 183 568 | 161 714 | 158 958 | 121 652 | 50 841787 | 418 | 97.88 | 93.27 | 50.96 | 66.27 |
| 07.03.19 | Autumn.2019 | 176 178 | 154 022 | 151 623 | 116 147 | 48 240246 | 415 | 97.91 | 93.37 | 51.00 | 65.93 |
| 23.03.19 | Autumn.2019 | 182 594 | 163 145 | 160 825 | 119 649 | 49 323363 | 412 | 98.07 | 93.69 | 50.57 | 65.53 |
| 23.03.19 | Autumn.2019 | 178 506 | 158 592 | 156 205 | 106 93 | 44 073003 | 412 | 97.94 | 93.37 | 49.57 | 59.90 |
| 23.03.19 | Autumn.2019 | 181 929 | 161 113 | 158 94 | 114 493 | 47 267738 | 413 | 98.02 | 93.56 | 50.03 | 62.93 |
| 23.03.19 | Autumn.2019 | 177 302 | 155 17 | 152 992 | 111 594 | 45 965053 | 412 | 98.04 | 93.63 | 50.03 | 62.94 |
| 23.03.19 | Autumn.2019 | 177 67 | 156 386 | 154 114 | 114 689 | 47 348544 | 413 | 98.03 | 93.65 | 50.19 | 64.55 |
| 23.03.19 | Autumn.2019 | 183 82 | 162 215 | 159 574 | 114 106 | 47 217768 | 414 | 97.97 | 93.49 | 50.12 | 62.07 |
| 30.03.19 | Autumn.2019 | 175 936 | 156 191 | 153 862 | 98 993 | 40 873695 | 413 | 98.00 | 93.53 | 50.04 | 56.27 |
| 30.03.19 | Autumn.2019 | 187 182 | 167 696 | 165 105 | 112 046 | 46 430401 | 414 | 97.98 | 93.46 | 50.09 | 59.86 |
| 30.03.19 | Autumn.2019 | 186 8 | 166 328 | 163 801 | 107 031 | 44 314330 | 414 | 97.96 | 93.38 | 50.13 | 57.30 |
| 30.03.19 | Autumn.2019 | 188 257 | 170 509 | 167 764 | 138 517 | 57 168660 | 413 | 98.23 | 94.18 | 50.15 | 73.58 |
| 30.03.19 | Autumn.2019 | 183 548 | 163 382 | 161 061 | 112 506 | 46 640293 | 415 | 98.02 | 93.57 | 49.65 | 61.30 |
| 31.10.19 | Spring.2019 | 180 743 | 159 952 | 157 484 | 132 881 | 55 659109 | 419 | 98.01 | 93.56 | 49.00 | 73.52 |
| 31.10.19 | Spring.2019 | 177 321 | 156 519 | 153 993 | 124 4 | 52 153046 | 419 | 97.98 | 93.46 | 48.95 | 70.16 |
| 21.12.19 | Summer.2020 | 175 712 | 153 198 | 150 676 | 113 801 | 47 477501 | 417 | 97.90 | 93.36 | 48.85 | 64.77 |
| 21.12.19 | Summer.2020 | 181 613 | 156 826 | 154 301 | 115 958 | 48 420255 | 418 | 97.84 | 93.15 | 48.89 | 63.85 |
| 23.01.20 | Summer.2020 | 170 055 | 148 372 | 146 178 | 111 78 | 46 121202 | 413 | 98.07 | 93.78 | 50.59 | 65.73 |
| 23.01.20 | Summer.2020 | 170 764 | 152 75 | 150 676 | 116 356 | 47 981785 | 412 | 98.13 | 93.90 | 50.63 | 68.14 |
| 03.02.2020 | Summer.2020 | 183 865 | 162 699 | 159 987 | 122 403 | 51 083482 | 417 | 98.00 | 93.55 | 50.24 | 66.57 |
| 03.02.2020 | Summer.2020 | 174 054 | 153 821 | 151 431 | 106 063 | 44 360747 | 418 | 98.03 | 93.68 | 50.64 | 60.94 |
| 03.02.2020 | Summer.2020 | 172 643 | 152 435 | 149 972 | 114 088 | 47 640929 | 418 | 97.98 | 93.54 | 50.33 | 66.08 |
| 12.02.20 | Summer.2020 | 182 89 | 163 667 | 161 326 | 114 443 | 47 243218 | 413 | 98.03 | 93.59 | 49.36 | 62.57 |
| 12.02.20 | Summer.2020 | 174 413 | 155 159 | 152 898 | 111 776 | 46 331169 | 415 | 98.08 | 93.76 | 49.88 | 64.09 |
| 12.02.20 | Summer.2020 | 182 802 | 158 744 | 156 263 | 107 281 | 44 572014 | 415 | 97.96 | 93.53 | 50.13 | 58.69 |
| 12.02.20 | Summer.2020 | 180 059 | 160 034 | 157 691 | 121 976 | 50 476539 | 414 | 98.06 | 93.72 | 49.88 | 67.74 |
| 12.02.20 | Summer.2020 | 186 67 | 167 725 | 165 482 | 125 981 | 51 864568 | 412 | 98.14 | 93.91 | 50.11 | 67.49 |
| 12.02.20 | Summer.2020 | 189 671 | 167 786 | 165 172 | 124 114 | 51 680557 | 416 | 97.99 | 93.56 | 50.27 | 65.44 |
| 12.02.20 | Summer.2020 | 179 389 | 157 474 | 154 941 | 106 972 | 44 792698 | 419 | 97.99 | 93.54 | 50.73 | 59.63 |
| 12.02.20 | Summer.2020 | 182 87 | 162 118 | 159 593 | 109 832 | 45 522325 | 414 | 98.07 | 93.77 | 49.83 | 60.06 |
| 12.02.20 | Summer.2020 | 175 188 | 156 379 | 154 018 | 102 42 | 42 229340 | 412 | 98.12 | 93.90 | 49.99 | 58.46 |
| 19.02.2020 | Summer.2020 | 180 567 | 163 575 | 160 919 | 108 681 | 44 644988 | 411 | 97.86 | 93.15 | 49.43 | 60.19 |
| 19.02.2020 | Summer.2020 | 180 06 | 158 982 | 156 566 | 109 812 | 45 502318 | 414 | 98.03 | 93.60 | 49.93 | 60.99 |
| 19.02.2020 | Summer.2020 | 175 48 | 152 694 | 150 489 | 111 73 | 46 199188 | 413 | 98.06 | 93.76 | 50.36 | 63.67 |
| 19.02.2020 | Summer.2020 | 170 452 | 152 303 | 149 99 | 100 833 | 41 541677 | 412 | 97.95 | 93.44 | 49.69 | 59.16 |
| 31.03.20 | Autumn.2020 | 180 279 | 161 675 | 159 416 | 112 735 | 46 532 803 | 413 | 98.08 | 93.77 | 49.46 | 62.53 |
| 31.03.20 | Autumn.2020 | 173 142 | 155 059 | 152 444 | 107 332 | 44 284968 | 413 | 97.99 | 93.54 | 49.20 | 61.99 |
| 31.03.20 | Autumn.2020 | 177 23 | 158 591 | 156 12 | 108 439 | 44 853026 | 414 | 97.95 | 93.40 | 49.53 | 61.19 |
| 13.04.20 | Autumn.2020 | 179 75 | 159 227 | 156 908 | 99 711 | 41 236026 | 414 | 98.07 | 93.72 | 48.78 | 55.47 |
| 13.04.20 | Autumn.2020 | 186 549 | 165 853 | 163 43 | 111 388 | 46 126982 | 414 | 98.03 | 93.60 | 49.06 | 59.71 |
| 13.04.20 | Autumn.2020 | 187 881 | 166 121 | 163 766 | 109 481 | 45 194271 | 413 | 98.10 | 93.80 | 49.07 | 58.27 |
| 24.10.20 | Spring.2020 | 172 669 | 152 295 | 149 926 | 103 495 | 43 018361 | 416 | 98.01 | 93.59 | 49.47 | 59.94 |
| 24.10.20 | Spring.2020 | 183 437 | 164 071 | 161 678 | 104 297 | 43 307005 | 415 | 98.02 | 93.60 | 49.51 | 56.86 |
| 24.12.20 | Summer.2021 | 173 208 | 154 131 | 151 718 | 103 602 | 43 070658 | 416 | 97.99 | 93.54 | 49.63 | 59.81 |
| 24.12.20 | Summer.2021 | 170 309 | 152 883 | 150 721 | 100 671 | 41 702704 | 414 | 98.01 | 93.54 | 49.54 | 59.11 |
| 03.01.21 | Summer.2021 | 180 627 | 166 845 | 164 442 | 113 064 | 47 145379 | 417 | 98.22 | 94.14 | 50.02 | 62.60 |
| 04.01.21 | Summer.2021 | 171 54 | 153 717 | 151 485 | 105 342 | 43 623424 | 414 | 98.01 | 93.57 | 49.29 | 61.41 |
| 04.01.21 | Summer.2021 | 185 158 | 157 095 | 154 254 | 107 476 | 44 691339 | 416 | 97.75 | 93.01 | 49.60 | 58.05 |
| 17.02.21 | Summer.2021 | 173 993 | 155 581 | 153 362 | 110 239 | 45 657648 | 414 | 98.05 | 93.68 | 50.24 | 63.36 |
| 17.02.21 | Summer.2021 | 184 637 | 163 488 | 161 177 | 107 573 | 44 705789 | 416 | 98.12 | 93.87 | 50.20 | 58.26 |
| 17.02.21 | Summer.2021 | 177 363 | 156 318 | 153 749 | 99 177 | 41 181919 | 415 | 97.98 | 93.52 | 50.02 | 55.92 |
| 23.02.21 | Summer.2021 | 171 437 | 150 077 | 147 713 | 102 826 | 42 779460 | 416 | 97.82 | 92.97 | 50.07 | 59.98 |
| 23.02.21 | Summer.2021 | 188 312 | 166 669 | 163 978 | 113 349 | 47 011603 | 415 | 98.03 | 93.62 | 50.19 | 60.19 |
| 23.02.21 | Summer.2021 | 173 901 | 152 966 | 150 499 | 103 025 | 42 961420 | 417 | 97.95 | 93.45 | 50.11 | 59.24 |
| 04.03.21 | Autumn.2021 | 179 024 | 160 083 | 157 625 | 104 452 | 43 322289 | 415 | 98.02 | 93.60 | 50.10 | 58.35 |
| 04.03.21 | Autumn.2021 | 177 915 | 156 321 | 153 817 | 100 168 | 41 612306 | 415 | 97.96 | 93.47 | 50.29 | 56.30 |
| 04.03.21 | Autumn.2021 | 174 111 | 153 217 | 150 72 | 100 591 | 41 850125 | 416 | 97.97 | 93.50 | 50.35 | 57.77 |
| 07.03.21 | Autumn.2021 | 176 772 | 152 966 | 150 36 | 98 141 | 40 545467 | 413 | 97.94 | 93.45 | 50.01 | 55.52 |
| 07.03.21 | Autumn.2021 | 175 101 | 154 793 | 152 326 | 99 36 | 41 168210 | 414 | 97.99 | 93.56 | 49.79 | 56.74 |
| 24.03.21 | Autumn.2021 | 179 512 | 159 314 | 156 692 | 95 922 | 39 844664 | 415 | 97.99 | 93.44 | 50.11 | 53.43 |
| 24.03.21 | Autumn.2021 | 186 089 | 167 182 | 164 686 | 110 106 | 45 309548 | 412 | 97.90 | 93.20 | 49.70 | 59.17 |
| 24.03.21 | Autumn.2021 | 170 051 | 152 779 | 150 747 | 112 786 | 46 193002 | 410 | 98.12 | 93.83 | 49.95 | 66.32 |

**Table S3.** Differences in interseasonal taxa distribution during the 3 consecutive years (p-value<0.05).

| **Genus** | **2019** | **2020** | **2021** |
| --- | --- | --- | --- |
| *Polaribacter* | March vs February  March vs October  March vs December | February vs October | January vs February  January vs March  March vs February |
| *Sulfitobacter* | March vs October | January vs February  February vs April  February vs October  February vs December |  |
| SAR11 Clade Ia | March vs February  March vs October  March vs December | March vs February  February vs April  February vs October |  |
| *Colwellia* | March vs October |  |  |
| *Planktomarina* | March vs February | March vs February  February vs April |  |
| Flavobacterial NS5 clade | March vs February | March vs February  February vs April |  |
| *Pseudoalteromonas* | March vs October |  |  |
| *Vicingus* | March vs February |  | March vs February |
| *Yoonia* |  | February vs October  February vs December |  |
| *Marinoscillum* |  | March vs February  February vs April |  |
| *Bacillus* | March vs December | February vs April  February vs October  February vs December | January vs February  January vs March |
| *Amylibacter* | March vs December | March vs February  February vs April  February vs October | January vs February  January vs March |
| SAR92 clade | March vs February  March vs December | March vs February  February vs April | January vs February |
| *Ulvibacter* |  |  | January vs March |
| *Paraglaciecola* | March vs February  March vs October  March vs December | January vs February  February vs October | January vs March |
| SUP05 clade |  | February vs April  February vs October  March vs April |  |
| *Polaromonas* |  | February vs October  March vs April | January vs February |
| *Ascidiaceihabitans* | March vs February | March vs February  February vs April  March vs April | March vs February |
| PeM15 |  | March vs February  February vs April |  |
| *Subdoligranulum* | March vs October  March vs December |  |  |
| SAR11 Clade II | March vs February | March vs February  February vs April  February vs December  March vs April |  |
| SAR11 Clade III |  | March vs February  February vs April  March vs April |  |
| *Candidatus* Nitrosopumilus | March vs October | February vs April  February vs October |  |
| SAR86 clade | March vs October | February vs April  February vs October |  |
| *Pseudohongiella* |  | March vs February  February vs April | March vs February |
| *Bacteroides* | March vs October  March vs December |  |  |
| OM43 clade | March vs February | March vs February  February vs April  March vs April |  |
| Flavobacterial NS4 clade |  | February vs October |  |
| *Magnetospira* |  | March vs February  February vs April |  |
| *Psychrobacter* |  | February vs October |  |
| *Glaciecola* | March vs October  March vs December |  |  |

**3 References**

1. Alarcón-Schumacher, T., Guajardo-Leiva, S., Antón, J., & Díez, B. (2019). Elucidating viral communities during a phytoplankton bloom on the West Antarctic Peninsula. Frontiers in Microbiology, 10, 1014
2. Annett, A. L., Henley, S. F., Venables, H. J., Meredith, M. P., Clarke, A., & Ganeshram, R. S. (2017). Silica cycling and isotopic composition in northern Marguerite Bay on the rapidly-warming western Antarctic Peninsula. Deep Sea Research Part II: Topical Studies in Oceanography, 139, 132-142
3. Beidler, I., Steinke, N., Schulze, T. et al. Alpha-glucans from bacterial necromass indicate an intra-population loop within the marine carbon cycle. Nat Commun 15, 4048 (2024). https://doi.org/10.1038/s41467-024-48301-5
4. Biggs, T. E., Huisman, J., & Brussaard, C. P. (2021). Viral lysis modifies seasonal phytoplankton dynamics and carbon flow in the Southern Ocean. The ISME journal, 15(12), 3615-3622
5. Carvalho, F., Fitzsimmons, J. N., Couto, N., Waite, N., Gorbunov, M., Kohut, J., ... & Schofield, O. (2020). Testing the Canyon Hypothesis: Evaluating light and nutrient controls of phytoplankton growth in penguin foraging hotspots along the West Antarctic Peninsula. Limnology and Oceanography, 65(3), 455-470
6. Costas-Selas, C., Martínez-García, S., Logares, R., Hernández-Ruiz, M., & Teira, E. (2023). Role of Bacterial Community Composition as a Driver of the Small-Sized Phytoplankton Community Structure in a Productive Coastal System. Microbial Ecology. http://doi.org/10.1007/s00248-022-02125-2
7. Deppeler, S., Schulz, K. G., Hancock, A., Pascoe, P., McKinlay, J., & Davidson, A. (2020). Ocean acidification reduces growth and grazing impact of Antarctic heterotrophic nanoflagellates. Biogeosciences, 17(16), 4153-4171
8. Evans, C., Brandsma, J., Pond, D. W., Venables, H. J., Meredith, M. P., Witte, H. J., ... & Brussaard, C. P. (2017). Drivers of interannual variability in virioplankton abundance at the coastal western Antarctic peninsula and the potential effects of climate change. Environmental Microbiology, 19(2), 740-755
9. Janse, I., Van Rijssel, M., Gottschal, J. C., Lancelot, C., & Gieskes, W. W. (1996). Carbohydrates in the North Sea during spring blooms of Phaeocystis: a specific fingerprint. Aquatic microbial ecology, 10(1), 97-103
10. Lopez-Simon, J., Vila-Nistal, M., Rosenova, A., De Corte, D., Baltar, F., & Martinez-Garcia, M. (2023). Viruses under the Antarctic Ice Shelf are active and potentially involved in global nutrient cycles. Nature Communications, 14(1), 8295
11. Ratnarajah, L., Blain, S., Boyd, P. W., Fourquez, M., Obernosterer, I., & Tagliabue, A. (2021). Resource Colimitation Drives Competition Between Phytoplankton and Bacteria in the Southern Ocean. Geophysical Research Letters. https://doi.org/10.1029/2020GL088369
12. Sherrell, R. M., Annett, A. L., Fitzsimmons, J. N., Roccanova, V. J., & Meredith, M. P. (2018). A shallow bathtub ring of local sedimentary iron input maintains the Palmer Deep biological hotspot on the West Antarctic Peninsula shelf. Philosophical Transactions of the Royal Society A: Mathematical, Physical and Engineering Sciences, 376(2122), 20170171
